# Multivariate analyses of Ethiopian durum wheat revealed stable and high yielding genotypes

**DOI:** 10.1371/journal.pone.0273008

**Published:** 2022-08-17

**Authors:** Behailu Mulugeta, Kassahun Tesfaye, Mulatu Geleta, Eva Johansson, Teklehaimanot Hailesilassie, Cecilia Hammenhag, Faris Hailu, Rodomiro Ortiz

**Affiliations:** 1 Institute of Biotechnology, Addis Ababa University, Addis Ababa, Ethiopia; 2 Department of Plant Breeding, Swedish University of Agricultural Sciences, Alnarp, Sweden; 3 Sinana Agricultural Research Center, OARI, Bale-Robe, Ethiopia; 4 Ethiopian Biotechnology Institute, Addis Ababa, Ethiopia; 5 Department of Biology and Biotechnology, Wollo University, Dessie, Ethiopia; Isfahan University of Technology, ISLAMIC REPUBLIC OF IRAN

## Abstract

Improving crop adaptation and stability across diverse and changing environmental conditions is essential to increasing grain yield per unit area. In turn, this contributes to meeting the increasing global food demand. Nevertheless, a number of factors challenge the efficiency of crop improvement programs, of which genotype-by-environment interaction (GEI) is one of the major factors. This study aimed to evaluate the performance and phenotypic stability of 385 Ethiopian durum wheat landraces and 35 cultivars; assess the pattern of genotype by environment interaction (GEI) effect, and identify stable and high-yielding landraces or cultivars using the additive main effect and multiplicative interaction (AMMI) and genotype main effect plus genotype by environment interaction biplot (GGE-biplot). The experiment was laid out in an alpha lattice design with two replications at five test sites (Akaki, Chefe Donsa, Holeta, Kulumsa, and Sinana). The combined analysis of variance revealed highly significant effects (*P* ≤ 0.01) of environments (E), genotype (G), and GEI on a phenotypic variation of traits evaluated, including grain yield. For all traits, the amount of phenotypic variance and GEI explained by the GGE biplot was higher than in AMMI2, but both exhibited significant effects of E and GEI on the genotypes. The AMMI model identified G169, G420, G413, G139, G415, G416, G417, and G418 as stable genotypes across testing sites. Whereas, the GGE biplot identified G169, G420, G415, G139, G106, G412, G413, and G417 as both high-yielding and stable across test sites. Hence, genotypes identified as stable and high yielding in the present study could be used in a durum wheat breeding program aimed at identifying genes and molecular markers associated with the crop’s productivity traits as well as developing stable and high-yielding cultivars for use in East Africa and beyond.

## Introduction

Wheat (*Triticum spp*. L.) is one of the most important cereal crops having global production of over 700 million tones and provides 20% of the daily protein requirements, and calories for 4.5 billion people globally [1]. Wheat contributes significantly to food security by providing about one-fifth of the daily energy and protein required by humans [2]. Ethiopia is the largest producer of wheat in sub-Saharan Africa, where the crop is well adapted to diverse agro-ecological zones. In Ethiopia, wheat is mainly cultivated in the highlands and mid-altitude areas, ranging from 1500 m to 3200 m above sea level, at latitudes ranging from 6° to 14°N and longitudes ranging from 35° to 42°E. Among the cereal crops produced in Ethiopia, wheat ranks fourth in terms of area of production, covering 1.7 million hectares that produce 4.7 million tons of grain, which account for 13.4% and 15.2% of the total area and production volume of the crop, respectively [3].

In plant breeding, the development of stable and high-yielding cultivars is of paramount importance to attain a high yield that is independent of varied environmental influences [4–8]. However, plant breeding has often been challenged by biotic and abiotic stresses [9–13]. Thus, multi-environmental trials (METs) have been widely adopted to enable the selection of stable and high yielding genotypes [14–20]. The strategic and consistent use of METs was widely implemented worldwide by Dr. Norman E. Borlaug (the Nobel Peace Price Laureate, 1970) through the diverse international nursery sets distributed yearly by the Centro Internacional de Mejoramiento de Maíz y Trigo (CIMMYT, México) with the aim of improving grain yield. His work resulted primarily in the increased and improved local food supply in Latin America and Asia [21, 22]. Stable cultivars with high yields are of particular importance to farmers in the developing world who have limited access to agricultural inputs [23].

Conducting METs is particularly crucial for the estimation of genotypic stability because the magnitude of genotype-by-environment interactions (GEI) impacts the mean performance of genotypes across environments [24–26]. A thorough understanding of GEI complexity is crucial for plant breeders in order to optimize the performance of their materials. This is because it facilitates the selection of suitable genotypes with high and stable yields in diverse environments [27–29].

To evaluate genotype stability and GEI from MET data, various multivariate statistical methods have been developed. These include the additive main effect and multiplicative interaction (AMMI) model [30–33], and the genotype main effect plus genotype by environment interaction (GGE) biplot [24, 33–37]. In practice, these methods have been used to select superior and stable genotypes across diverse environments in various field crops, including durum wheat [16, 29, 38–44].

Ethiopia is considered as a secondary center of origin and diversity for tetraploid wheat species such as *Triticum ethiopicum* Jakubzo, *T*. *dicoccon* Schrank, *T*. *durum* Desf., *Triticum polonicum* L., *Triticum pyriamidale* Perc, *Triticum turgidum* L. *and Triticum compactum* Host [45–54]. Previous research has shown that the Ethiopian durum wheat gene pool contains unique alleles contributing to host plant resistance to pathogens, drought tolerance, and grain quality [47, 55–57]. Hence, the Ethiopian durum wheat germplasm can be considered an invaluable reservoir of economically useful genes for developing new durum wheat cultivars through breeding. Research on phenotypic stability and GEI targeting Ethiopian wheat germplasm has primarily focused on hexaploid wheat genotypes and cultivars [58–61]. Given the highly limited use of Ethiopian durum wheat germplasm in previous studies on genotype stability and GEI [62–64], information regarding the effects of GEI on its landraces and cultivars is scarce and insufficient.

The aim of the present study was to evaluate phenotypic stability and the magnitude of GEI for various phenotypic traits in a broad set of Ethiopian durum wheat landraces and cultivars through conducting METs in Ethiopia. Based on the results of the stability and GEI analyses, the study also sought to identify suitable genotypes that could be used in durum wheat breeding programs in order to enhance its production and productivity.

## Materials and methods

### Experimental environments and plant materials

**Fig 1:**
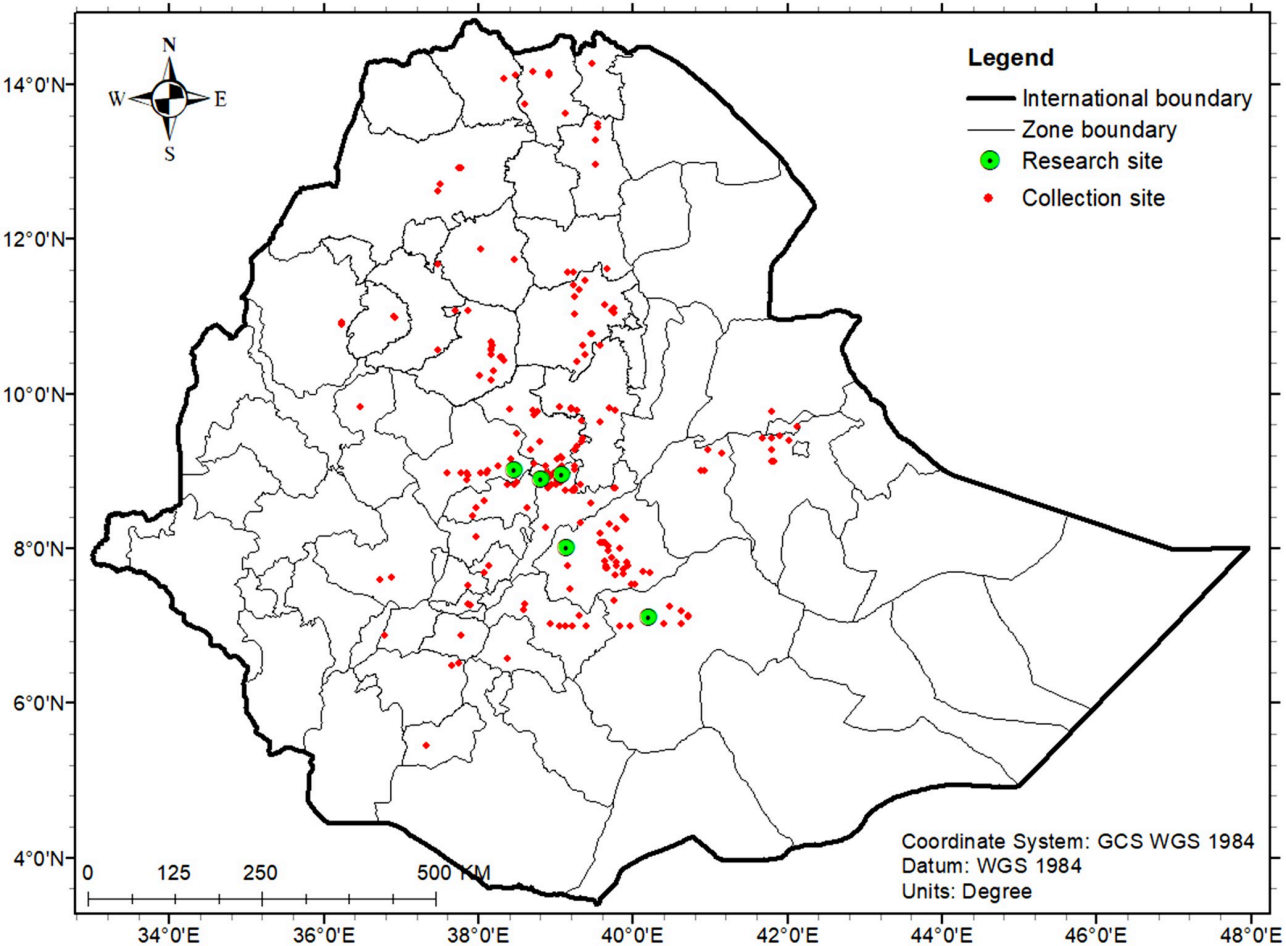
Map of Ethiopia showing the test sites (green circles) and the original germplasm collection sites of the landrace used in this study (red rhombus).

In this study, 420 Ethiopian durum wheat accessions comprising 385 landraces (G001–G385) and 35 cultivars (G386–G420) were evaluated under rainfed conditions during the 2019–2020 crop growing season in Ethiopia. The field trials were conducted at five sites known to produce durum wheat, namely Akaki, Chefe Donsa, Holeta, Kulumsa, and Sinana (S1 Table). These sites, which will be referred to as “test sites” represent different agro-ecozones in the country where wheat is grown under rainfed conditions (Table 1, Fig 1). The landraces used in this study were collected by the Ethiopian Biodiversity Institute (EBI) from areas in the country where durum wheat is cultivated as a major crop. Among the 35 cultivars, 10 were released at the regional and 25 at the national levels. Hereafter, the term ‘genotype’ will be used to refer to both the individual landraces and cultivars for the sake of simplicity.

**Table 1:** A description of the testing sites.

		Test sites				
		Sinana	Kulumsa	Chefe Donsa	Akaki	Holeta
Geographical description	Latitude (N)	07°06´57.58´´	08°01´10.28´´	08°58´56.55´´	09°53´47.62´´	09°01´14.62´´
	Longitude (E)	40°13´38.00´´	39°09´36.92´´	39°09´12.59´´	39°49´15.51´´	38°28´25.62´´
	Altitude (m.a.s.l.)	2400	2208	2360	2203	2230
Soil properties	pH	6.2	6.5	7.8	7.4	5.4
	Texture	Clay	Clay	Heavy Clay	Heavy Clay	Clay
	OMC (%)	3.9	3.6	3.7	3.4	3.1

m.a.s.l. = meter above sea level; OMC = Organic matter content

### Experimental layout and design

The genotypes were laid out in the field trials using the alpha lattice design [65] with two replications and they were assigned randomly to each replication with the aid of R software. In each plot, seeds were planted in two rows of 2.5 m each, separated by 0.2 m. Fertilizers were applied to each plot at a rate of 50 kg ha^-1^ of N and 100 kg ha^-1^ of P_2_O_5_ at the planting and jointing stage. Other agronomic practices were treated as non-experimental variables and applied uniformly to the entire experimental area.

### Measurement of phenotypic traits

Data for eight different traits, representing morpho-phenological (days to heading and days to maturity), morpho-agronomical (plant height, spike length, and the number of effective tillers per plant), grain yield, and grain yield-related traits (numbers of spikelets per spike, thousand kernel weight), were measured according to the wheat descriptors [66]. Days to heading was scored as the number of days it takes from planting to the heading of 50% of the plants on a plot. Days to maturity was scored as the number of days it takes from planting to the physiological maturity of 75% of plants on a plot. The harvested grains were dried at room temperature until their moisture content reached the standard 12%, as determined by using a digital grain moisture meter. This was followed by determining grain yield per hectare and thousand kernel weight. The weight of clean seeds harvested from the whole plot was used to estimate grain yield per hectare. Thousand kernel weight was calculated as weight in grams of 1000 grains. For traits scored at individual plant level, five plants per plot were randomly selected and tagged with a red string to collect phenotypic data. The traits scored for each of the five plants were; the number of effective tillers per plant (as the number of spike-bearing tillers per plant), plant height (a height in cm of the plant before maturity), spike length (a length from the tip of the spike without awns to the pedicule base, in cm), and the number of spikelets per spike (as an average number of spikelets per spike from all plants considered).

### Phenotypic data analysis

#### Analysis of variance

The combined analysis of variance was performed using SAS software version 9.4 to evaluate the GEI and to partition the total variation into the variation due to G, E, and GEI. A Bartlett’s test was performed in R to check the homogeneity of the error variance. To determine GEI, genotypes were treated as fixed effects and test sites as random effects. The stability parameters were analysed using the R program version 4.0. The AMMI and GGE-biplot analyses were conducted with Genstat software version 18.0.

#### Multivariate stability analysis

The multivariate stability analysis AMMI [32] and GGE biplot [37] were used to investigate the stability of the genotypes across testing environments.

#### AMMI analysis

The AMMI model was used to reveal the patterns of GEI [31] as per the below equation:-

$${\text{Y}}_{\text{ij}}=\mu +{\text{G}}_{\text{i}}+{\text{E}}_{\text{j}}+\sum_{\text{K}=1}^{\text{N}}\;\lambda_{\text{k}}\gamma_{\text{ik}}\delta_{\text{jk}}+\rho_{\text{ij}}+\varepsilon_{\text{ij}}$$ (1)

where Y_ij_ is the yield of i^th^ genotype in the j^th^ environment; μ is grand mean; G_i_ and E_j_ are the genotypes and environment deviation from grand mean, respectively; λ_k_ is the singular value for k^th^ IPC (interaction principal component); γ_ik_ is the genotype G eigenvector (score) for k^th^ IPC axis; δ_jk_ is the environment E eigenvector (score) for k^th^ IPC axis; ρ_ij_ is the interaction residual; N is the number of principal components in the model and ε_ij_ is the random error.

The AMMI biplot determines the stability of genotypes based on their interaction principal component analyses (IPCA) scores, as described by Carbonell et al. [67]. Genotypes close to the origin of the AMMI biplot have low GEI and are hence considered to have wide adaptation across environments with lower mean performance.

Univariate stability parameters such as yield stability index (YSi), rank yield stability index (rYSi), AMMI stability value (ASV), and AMMI rank stability value (rASV) were estimated to evaluate the stability of genotypes.

The AMMI’s stability value (ASV) was calculated following Purchase et al. [68] as:-

$$\text{ASV}=\sqrt{{\left[\left(\frac{{\text{SSIPC}}_{1}}{{\text{SSIPC}}_{2}}\right)*{\text{IPCA}}_{1}\right]}^{2}+{\text{IPCA}}_{2}{}^{2}}$$ (2)

where SSIPC_1_/SSIPC_2_ is the weight given to the IPCA1 value by dividing the sum square of interaction principal component one (SSIPC1) by the sum square of interaction principal component two (SSIPC2); and the IPCA1 and IPCA2 scores are the genotypic scores in the AMMI model.

The yield stability index (YSI) was calculated according to Kang [69] for simultaneous selection of genotypes based on their yield and stability as follows:-

YSI=RASV+RY (3)

where RASV is the rank of the AMMI stability value and RY is the rank of the mean grain yield of a genotype across sites.

#### GGE biplot analysis

The GGE biplot analysis was carried out based on singular value decomposition (SVD) following the model by Yan and Tinker [70] as:-

$$\bar{\text{Y}}\text{ij}=\lambda_{1}\gamma_{i1}\delta_{\text{j}1}+\lambda_{2}\gamma_{i2}\delta_{\text{j}2}+\xi_{\text{ij}}$$ (4)

where $\bar{\text{Y}}\text{ij}$ is the mean of genotype *i* in test site *j*; λ_1_ and λ_2_ are the SVD of the first and second principal components (PC_1_ and PC_2_), respectively, associated with the matrix of the effects of a genotype contributing to the effects of GEI; γ_i1_ and γ_j2_ are eigenvectors of the PC1 and PC2 associated with the effects of genotype i, respectively; δ_j1_ and δ_j2_ are eigenvectors of the PC1 and PC2 associated with the effects of test site j, respectively and ξij is the residual of the model associated with genotype i in the environment j.

The cosine of the angle with respect to the position of a test site and the genotypes on the GGE biplot determine the mean performance of the genotypes at that specific environment.“Which won where” polygon view of GGE biplot was also constructed in this study, as it is an effective tool to investigate and visualize mega-environments [35]. However, considering that a mega-environment is a group of geographical locations that consistently exhibit the same best genotypes year after year in field trial [71], our analysis based on data from one-year field trials is only indicative of the existence of mega-environments. Thus, the suggestive "mega-environments" revealed in this study are referred to as "environment-groups" in order to distinguish them from the mega-environments that would be defined based on multi-year field trials. The lines drawn from the origin are perpendicular to the polygon sides and make sectors [72]. For the analysis of the GGE biplot in this study, the data were not transformed (“Transform = 0”), not scaled (“Scaling = 0”), and were environment-centered (“Centering = 2”). The biplot was based on environment-focused singular value partitioning (“SVP = 2”) and therefore is appropriate for visualizing the relationships among environments.

## Results

### Genotype and environment effects on the phenotypic traits

The analysis of variance revealed a highly significant (*P≤0*.*01*) effect of G, E, and GEI for all evaluated phenotypic traits except that GEI was not significant for the number of effective tillers per plant (Table 2). In addition, replication and block also had significant effects on phenotypic traits (*P≤0*.*01*) (Table 2), which could be attributed to factors such as soil variations within a test site. The AMMI analysis also revealed the significant effects of G, E, and GEI for all phenotypic traits analyzed (Table 3). Among the three sources of phenotypic variances (E,G, and GEI), variance due to E was the largest across all traits, indicating its prominence in the determination of phenotypic values (Table 2). Additionally, E was the source of more than a quarter of the total sum of squares in all target traits, ranging from 25.8% (for thousand kernel weight) to 76.1% (for plant height) (Table 3), also indicating the importance of the cultivation environments for all targeted traits.

**Table 2:** Mean square (MS) and estimated variance (EV) for each source of variation of eight traits across 420 Ethiopian durum wheat genotypes tested at five environments during the main cropping season of 2019–2020.

Source of variation	DF	Days to heading		Days to maturity		Plant height (cm)		Spike length (cm)		Numbers of spikelets per spike		Number of effective tillers per plant		Thousand kernel weight (g)		Grain yield (t ha^-1^)	
		MS	EV	MS	EV	MS	EV	MS	EV	MS	EV	MS	EV	MS	EV	MS	EV
Location	4	15500	18.3	6.5x10^3^	7.4	25x10^3^	299.3	5.0x10^3^	1.3	5.2x10^3^	5.9	532.8	0.6	8.4x10^3^	19.8	321 x10^3^	368.5
		***		***		***		***		***		***		***		***	
Replication	1	448.2	0.0	619.5	0.1	6.6x10^3^	2.6	17.1	0.1	17.1	0.0	860.5	0.4	301.2	0.0	4.6 x10^3^	0.0
		***		***		***		***		***		***		***		ns	
Block(Adj.)	40	267.4	2.2	36.6	0.2	695.2	4.7	14.1	0.1	14.1	0.1	19.6	0.2	282.1	2.0	3.8x10^3^	32.3
		***		***		***		***		***		***		***		***	
Replication (Location)	4	242.0	0.56	316.0	0.7	755.9	1.6	41.2	0.1	41.2	0.1	41.6	0.1	169.0	0.4	12.1x10^3^	28.4
		***		***		***		***		***		***		***		***	
Genotype(Adj.)	419	84.7	8.0	18.0	0.9	370.6	30.3	9.7	0.85	9.7	0.76	4.0	0.2	142.6	13.4	834.1	56.4
		***		***		***		***		***		***		***		***	
Genotype * Environment	1676	9.4	1.3	9.2	1.3	83.3	8.	2.5	0.2	2.5	0.3	2.2	0.00	15.7	4.1	300.3	39.0
		***		***		***		***		***		*ns*		***		***	
Error	2055	6.7	6.70	6.1	6.5	66.4	66.4	1.9	1.1	1.9	1.9	2.2	2.1	7.6	7.6	222.4	222.4
R^2 †^		0.9		0.8		0.9		0.8		0.9		0.7		0.9		0.8	
CV (%)		3.7		2.0		8.5		13.3		8.0		19.2		8.9		18.1	
RMSE		2.7		2.7		8.2		1.1		1.4		1.5		2.8		15.7	
$\bar{\text{x}}$		73.0		136.0		96.3		8.0		17.0		6.0		40.9		7.0	

** = highly significant (*P* < 0.01); ns = not significant (P > *0*.*05*); Adj. = adjusted, DF = Degrees of freedom; R^2^ = coefficient of determination; CV = coefficient of variation; RMSE = Root mean squared error

**Table 3:** Mean square (MS), percentage of the total sum of squares (PTSS) from AMMI analysis of variance for eight traits in 420 Ethiopian durum wheat genotypes grown in five environments, and percentage of total interaction sum of squares (PTISS) of the interaction principal components (IPC).

Source of Variation	DF	Days to heading			Days to maturity			Plant height			Number of effective tillers per plant		
		MS	PTSS	PTISS	MS	PTSS	PTISS	MS	PTSS	PTISS	MS	PTSS	PTISS
ENV	4	15594.4**	50.7		6527.5**	52.2		252192.2**	76.1		532.8**	27.8	
GEN	419	107.7**	36.7		20.5**	17.2		424.8**	13.4		4.5**	24.8	
GEN*ENV	1676	9.3**	12.7		9.2**	30.7		83.3**	10.5		2.2^ns^	47.3	
IPCA1	422	23.4**		66.71	14.9**		43.5	108.8**		34.9	3.0**		43.1
IPCA2	420	11.3**		32.13	8.9**		26.2	85.8**		26.2	2.4**		34.0
IPCA3	418	0.4**		1.16	5.9^ns^		17.2	74.6**		24.9	1.6^ns^		22.9
IPCA4	416				4.5^ns^		13.2	44.2^ns^		14.0	0.0^ns^		0.01
Error	2095	7			6.5			68			2.37		
Source of Variation	DF	Spike length			Number of spikelets per spike			Thousand kernel weight			Grain yield		
		MS	PTSS	PTISS	MS	PTSS	PTISS	MS	PTSS	PTISS	MS	PTSS	PTISS
ENV	4	1138.7**	38.8		5023.7**	70.7		8400.3**	25.8		3213.1**	57.3	
GEN	419	11.1**	39.5		10.1**	14.9		167.5**	54.0		10.9**	20.3	
GEN*ENV	1676	1.5**	21.7		2.5**	14.5		15.9**	20.2		3.01**	22.5	
IPCA1	422	2.2**		39.2	4.2**		49.0	33.4**		55.57	5.5**		47.3
IPCA2	420	1.5**		29.4	2.5**		26.5	16.3**		26.81	3.0**		28.9
IPCA3	418	1.0^ns^		18.9	2.1**		23.3	10.2**		16.85	2.7**		23.3
IPCA4	416	0.7^ns^		12.5	0.01^ns^		1.3	0.01^ns^		0.76	0.1^ns^		1.0
Error	2095	1.10			2.0			7.8			2.4		

** = highly significant (*P* < 0.01);

ns = not significant (P > *0*.*05*); ENV = Environment; GEN = Genotype; DF = Degrees of freedom

### Impacts of the environments on the performance of genotypes

There was a large variation in the mean grain yield of genotypes per location, with 3.5 t ha^-1^ for genotype G277 at Akaki to 11 t ha^-1^ for genotype G413 at Chefe Donsa. The overall mean performance of the genotypes varied across the five test sites with the highest and lowest mean grain yield obtained at Chefe Donsa (8.4 t ha^-1^) and Holeta (4.3 t ha^-1^), respectively (Table 4). The performance of the other target traits also differed across the test sites. For example, the number of spikelets per spike ranged from 14 (at Holeta) to 21 (at Sinana); spike length ranged from 6.25 (at Akaki) to 9.5 (at Sinana); the number of effective tillers per plant ranged from 4 (at Holeta) to 7 (at Sinana); and plant height ranged from 69.5 (at Akaki) to 117.1 (at Sinana; Table 4).

**Table 4:** Test site-specific mean values, IPCA1 score, and the first four genotypes selected by AMMI for the eight traits: Grain yield (t ha-1), plant height (cm), spike length (cm), number of effective tillers per plant, number of spikelets per spike, thousand kernel weight (g), days to heading and maturity.

Traits	Environments	Mean	IPCA_1_**	1^st^	2^nd^	3^rd^	4^th^
Grain yield	Sinana	8.2	-5.08	G292	G390	G393	G397
	Kulumsa	7.8	1.93	G169	G420	G102	G279
	Chefe Donsa	8.4	1.55	G413	G416	G415	G151
	Holeta	4.3	0.00	G413	G416	G415	G393
	Akaki	4.9	1.60	G169	G420	G413	G415
Plant height	Sinana	117.1	9.93	G152	G32	G277	G275
	Kulumsa	101.3	0.73	G032	G316	G287	G263
	Chefe Donsa	100.4	-0.25	G032	G282	G277	G287
	Akaki	69.5	-4.08	G388	G032	G409	G197
	Holeta	93.4	-6.33	G032	G022	G197	G316
Spike length	Sinana	9.5	2.94	G090	G103	G190	G385
	Holeta	8.2	1.86	G064	G063	G027	G384
	Kulumsa	8.1	-0.51	G307	G293	G298	G308
	Akaki	6.3	-1.65	G004	G135	G342	G062
	Chefe Donsa	7.7	-2.69	G235	G412	G284	G272
Number of effective tillers per plant	Chefe Donsa	6.5	3.52	G313	G288	G297	G212
	Holeta	4.8	1.20	G006	G128	G085	G069
	Kulumsa	6.1	0.18	G088	G008	G001	G276
	Akaki	6.5	-1.57	G088	G183	G001	G307
	Sinana	6.9	-3.32	G183	G364	G088	G313
Numbers of spikelets per spike	Sinana	20.9	1.72	G304	G272	G118	G302
	Holeta	14.5	1.68	G304	G302	G315	G306
	Kulumsa	18.2	1.64	G315	G306	G304	G302
	Chefe Donsa	18.2	-0.20	G360	G384	G310	G351
	Akaki	15.8	-4.83	G058	G247	G155	G065
Thousand kernel weight	Akaki	35.2	7.44	G355	G418	G172	G391
	Chefe Donsa	42.7	1.42	G390	G365	G353	G418
	Sinana	42.1	-1.95	G389	G410	G009	G392
	Holeta	42.2	-2.95	G410	G418	G392	G400
	Kulumsa	42.2	-3.95	G410	G265	G418	G166
Days to heading	Akaki	77.3	6.33	G315	G103	G352	G306
	Holeta	74.9	0.80	G134	G202	G239	G147
	Chefe Donsa	74.9	0.76	G202	G239	G147	G58
	Kulumsa	68.01	-3.50	G306	G108	G79G	G196
	Sinana	68.6	-3.94	G79	G306	G196	G79
Days to Maturity	Kulumsa	133.5	6.21	G316	G263	G032	G277
	Holeta	132.6	-0.33	G263	G197	G316	G058
	Sinana	138.2	-0.46	G286	G032	G196	G283
	Chefe Donsa	137.4	-1.27	G277	G197	G196	G275
	Akaki	138.6	-4.14	G300	G276	G274	G058

**Fig 2:**
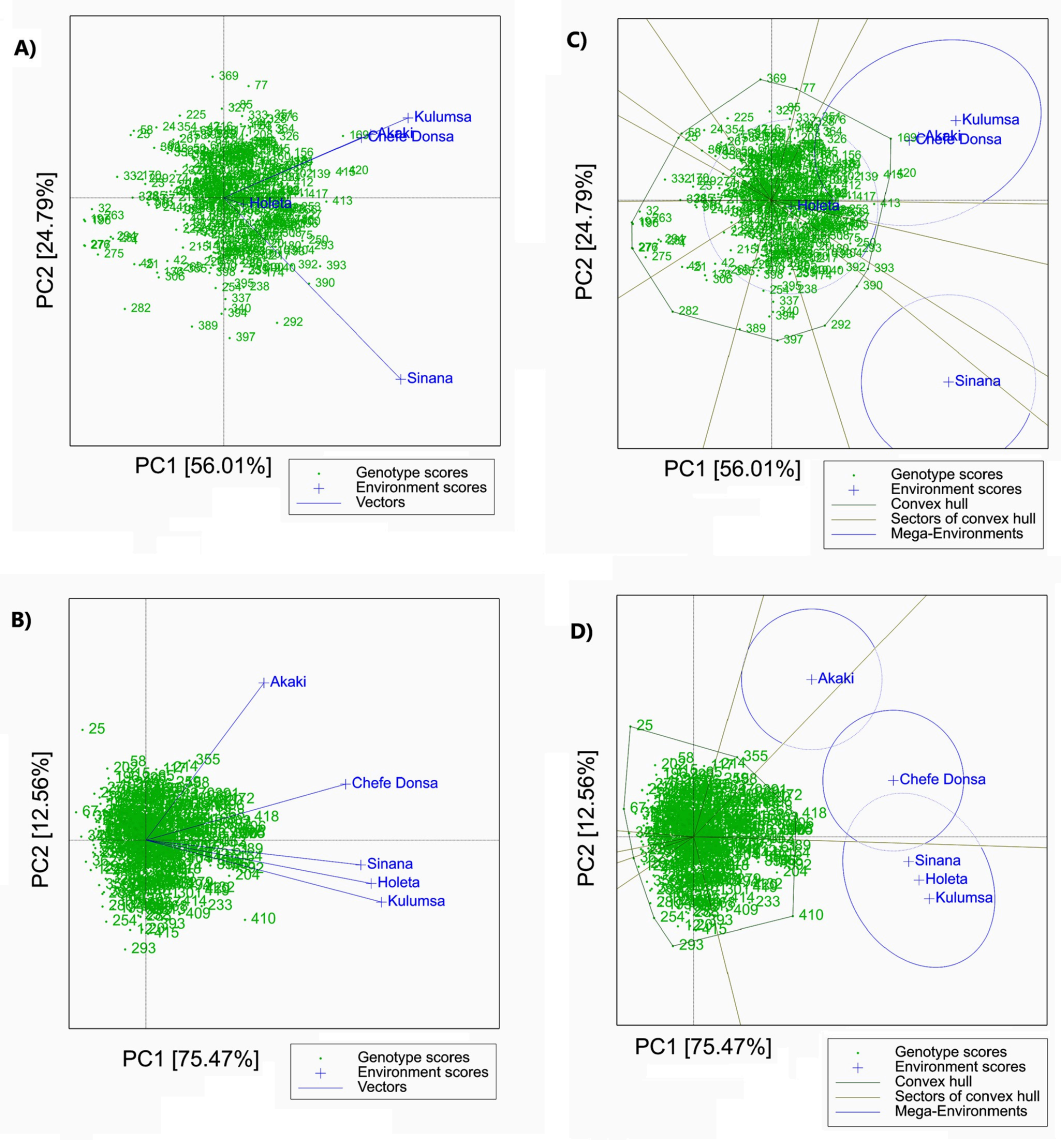
GGE-biplot for grain yield (A) and thousand-kernel weight (B), and polygon view of the GGE-biplot showing “which win where” pattern for grain yield (C) and thousand-kernel weight (D) under the five test sites.

**Fig 3:**
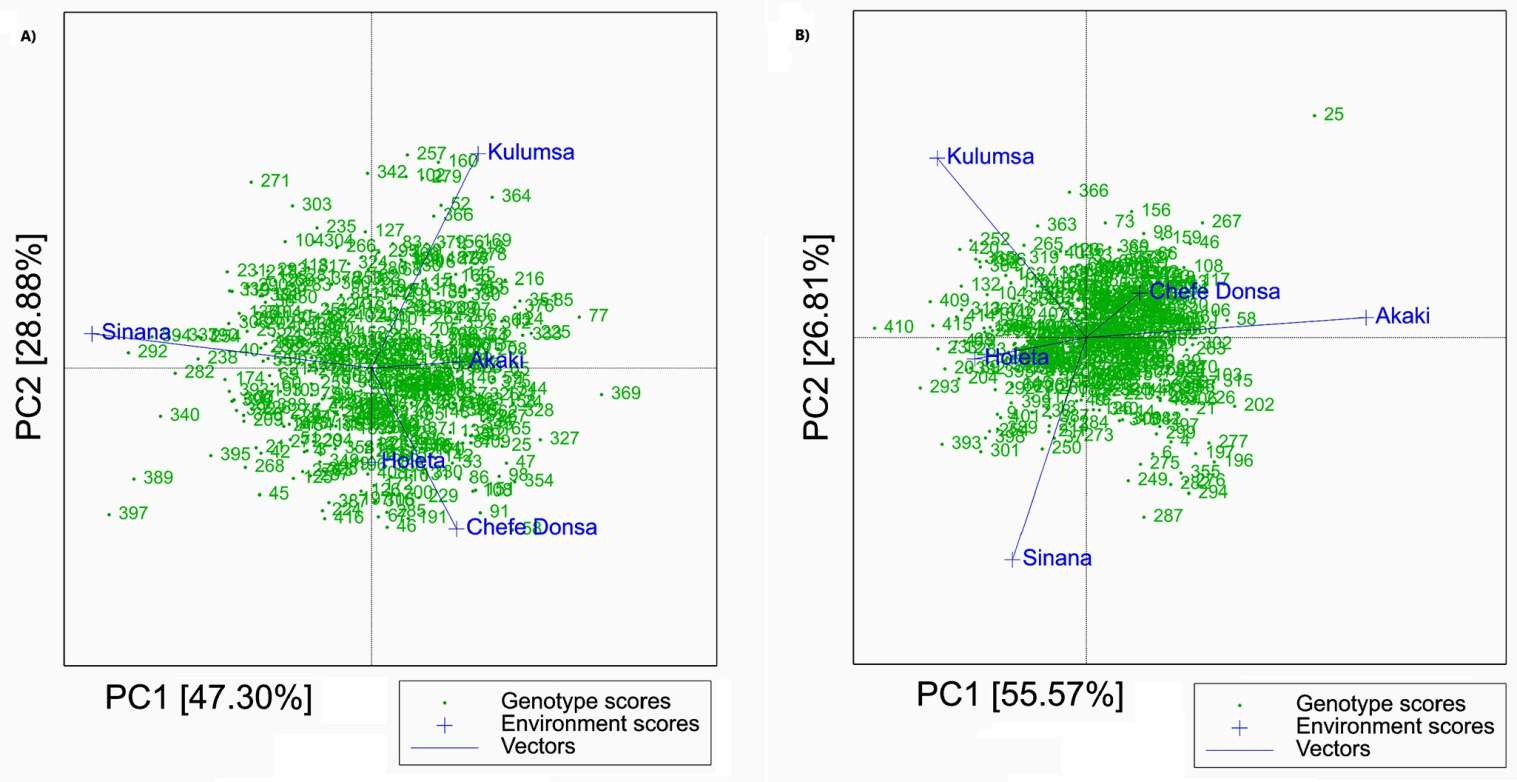
Additive main effect multiplicative model 2 (AMMI2) biplot for grain yield (A) and thousand-kernel weight (B) for 420 durum wheat genotypes and five test sites, respectively.

The AMMI results indicate that for grain yield, spike length, the number of effective tillers per plant, and plant height, Sinana had the highest IPCA1 (either positive or negative) and the lowest IPCA2 among the five test sites. By definition [67], this makes Sinana a suitable test site for discriminating genotypes based on these traits. These results were further verified through the GGE biplot model, where Sinana showed the highest discriminating power for grain yield among the test sites as shown by the length of the environment vector (Fig 2A). At Holeta test site, the AMMI2 biplot revealed low IPCA1 and IPCA2 for grain yield (Fig 3A) and thousand-kernel weight (Fig 3B). By definition [67, 72], low IPCA values for a given test environment indicate low interactions of the genotypes with that test site. Hence, the results of this study indicate low GEI for both grain yield and thousand kernel weight at Holeta (Fig 3A and 3B). This is consistent with the findings of the GGE biplot model, which showed that Holeta was the least discriminating and least informative test site for grain yield (Fig 2A). For thousand-kernel weight, however, the GGE biplot model did not clearly differentiate the test sites in terms of their discriminating abilities and their GEI impacts (Fig 2B).

**Fig 4:**
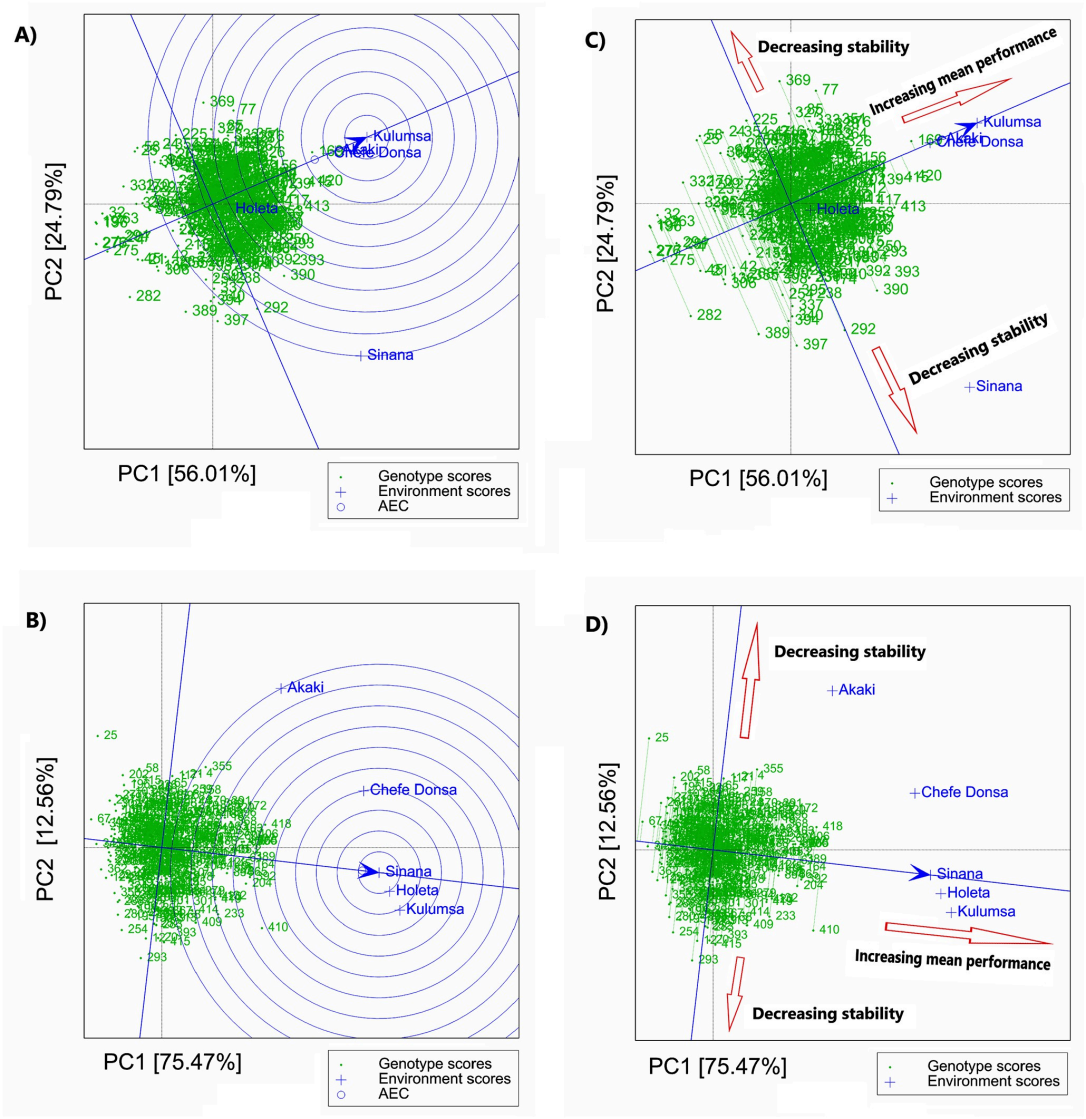
GGE-biplot showing the average environment coordination (AEC) based on the environment-focused scaling for grain yield (A) and thousand kernel weight (B), and the mean performance and stability of genotypes for grain yield at Kulumsa (C) and for thousand kernel weight at Sinana (D).

Among the five test sites, Akaki, Chefe Donsa, and Kulumsa had small angles with the Average Environment Axis (AEA; Fig 4A) in the GGE biplot for grain yield, which indicate that they are representative environments for genotype evaluations. Similarly, Sinana, Holeta and Kulumsa had small angles with the AEA in the GGE biplot for thousand-kernel weight, and hence they are respresenative environment for this trait (Fig 4B).

### GEI effects on phenotypic traits

The analyses of variance revealed a significant GEI effects on all traits except for the number of effective tillers per plant (Tables 2 and 3). The GEI explained 10 to 48% of the total variation in the different traits, as shown by the PTSS values from the AMMI analyses (Table 3). Furthermore, IPCA1 and IPCA2 obtained from the AMMI model were highly significant for all traits and they jointly explained 61 to 99% of the total variation, as measured by the percentage of the total interaction sum of squares (PTISS; Table 3). Environmental (soil) variation within each test site (Table 2) as well as genotypic variation within accessions (given that they are landraces) most likely contributed to the high GEI levels recorded in this study. In addition, the fact that the best genotypes differed among test sites provided further evidence of the large effects of GEI.

The AMMI analysis was used to identify the top four genotypes for all phenotypic traits per test site. In most cases, the top four genotypes for a given trait differed among the test sites (Table 4). However, for grain yield, six genotypes (G393, G169, G420, G413, G416, and G415) were among the top four genotypes in at least two of the test sites. Sinana was the most distinct test site for grain yield, as it differed from the other test sites in three of the four top-ranking genotypes (Table 4).

The GGE biplot, with PC1 and PC2 jointly explaining 80.8% of the total variation (G+GE), further confirmed the strong effect of GEI on grain yield. The polygon view of the GGE biplot identified G169, G420, G393, G390, G292, G025, G058, G276, G390, G397, G389, G282, G077, and G196 as the most responsive genotypes to the environmental interaction for grain yield, as they were placed on the vertices of the polygon. The genotypes that were placed within the polygon are comparatively less responsive to the environmental interaction (Fig 2C). The polygon view of the GGE biplot for grain yield grouped the test sites into three environment-groups (potential mega-environments) with Akaki, Chefe Donsa, and Kulunsa are included in the first environment-groups, while Sinana and Holeta included in the second and third environment-groups, respectively (Fig 2C). According to the polygon view, the winning genotypes in the first environment-groups was G169, whereas G390 was the winning genotypes in the second environment-groups (Fig 2C).

For thousand kernel weight, plant height, spike length, and the number of spikelets per spike, the GGE biplot revealed 88.0%, 73.5%, 81.4% and 76.8% of total G+GE, respectively. The environment-groups for thousand kernel weight differed from that of grain yield, as the first environment-groups for thousand kernel weight contained Sinana, Kulumsa and Holeta, while the second and the third environment-groups included Akaki and Chefe Donsa, respectively (Fig 2D). The winning genotypes for thousand kernel weight at the first environment-groups were G410, G418, G389, and G392. In the second environment-groups, G418 and G172 were the winning genotypes, whereas G355 was the winning genotype in the third environment-groups (Fig 2D).

### Stability of genotypes

The AMMI analysis is commonly used to depict high-performing and stable genotypes, where an IPCA1 value close to zero is used as a measure of stability. In this study, several high yielding genotypes across test sites were revealed to have low IPCA1 values (between -0.2 and 0.2), including G413, G420, G415, G293, G102, G412, G266, G207 and G075 (Table 5). The AMMI stability value (ASV) for grain yield was computed for all genotypes, and was found to vary for different genotypes ranging from 0.01 to 1.41 (Table 5). In principle, a low ASV represents a high genotypic stability across test sites, and ASV below 0.2 was recorded for G417, G411, G410, G096, G022, and G244. The analysis of the yield stability index (YSI) revealed G415, G417, G411, G412, and G410 as the most stable genotypes having YSI of below 100. Genotypes with a low YSI, partially overlapped with those having a low ASV indicating their stability across test sites (Table 5).

**Table 5:** The top and bottom 10% of the 420 genotypes in mean grain yield across the five cultivation environments (GY (t ha^-1^)) and their corresponding AMMI stability value (ASV), yield stability index (YSI), rank ASV (rASV) and rank YSI(rYSI), as well as their first three interaction principal components (IPC1 –IPC3).

Top 10% Best performing and stable genotypes (Top-down)										Bottom 10% least performing and un-stable (From bottom-up)									
S/N	Genotypes	GY	ASV	YSI	rASV	rYSI	IPCA_1_	IPCA_2_	IPCA_3_	S/N	Genotypes	GY	ASV	YSI	rASV	rYSI	IPCA_1_	IPCA_2_	IPCA_3_
1	G169	9.79	0.74	363	362	1	-0.28	0.52	0.32	42	G007	5.60	0.40	566	187	379	-0.02	0.58	-0.59
2	G413	9.78	0.30	128	126	2	0.17	-0.48	0.30	41	G182	5.57	0.21	450	70	380	0.03	-0.05	-0.14
3	G420	9.72	0.42	210	207	3	0.15	0.30	0.54	40	G022	5.53	0.18	435	54	381	-0.08	-0.10	-0.50
4	G415	9.43	0.21	72	68	4	0.09	-0.12	0.57	39	G237	5.53	0.35	539	158	381	-0.17	-0.26	-0.17
5	G393	9.09	0.83	392	387	5	0.60	-0.17	0.71	38	G267	5.51	0.70	731	348	383	-0.56	-0.06	0.04
6	G390	8.91	1.01	415	409	6	0.86	0.02	0.22	37	G091	5.48	0.81	767	383	384	-0.63	-0.39	-0.23
7	G416	8.79	0.60	316	309	7	0.24	-0.54	0.27	36	G362	5.45	0.64	711	326	385	-0.53	-0.06	-0.96
8	G417	8.76	0.03	12	4	8	0.25	-0.27	0.39	35	G042	5.39	0.71	738	352	386	0.44	-0.55	0.32
9	G293	8.70	0.70	354	345	9	-0.01	0.47	0.29	34	G268	5.39	0.82	771	384	387	0.34	-0.39	-0.33
10	G139	8.70	0.45	234	224	10	0.49	0.29	0.42	33	G236	5.38	0.20	453	65	388	-0.16	-0.09	-0.22
11	G253	8.55	0.35	172	161	11	0.23	0.20	0.16	32	G350	5.36	0.53	654	265	389	-0.36	-0.09	-0.28
12	G392	8.53	0.80	392	380	12	0.51	-0.08	0.18	31	G108	5.34	0.73	749	359	390	-0.51	-0.57	-0.07
13	G411	8.49	0.01	14	1	13	0.23	-0.20	0.18	30	G048	5.34	0.22	471	80	391	-0.15	-0.33	-0.23
14	G250	8.48	0.60	318	304	14	0.52	0.14	0.56	29	G067	5.14	0.53	658	266	392	-0.59	-0.20	-0.41
15	G371	8.39	0.35	171	156	15	-0.27	-0.16	0.48	28	G274	5.13	0.25	481	88	393	-0.20	-0.01	-0.13
16	G088	8.38	0.31	149	133	16	0.57	0.30	0.12	27	G024	5.12	0.83	783	389	394	-0.14	-0.53	-0.17
17	G404	8.37	0.66	350	333	17	0.27	0.21	0.12	26	G136	5.08	0.80	773	378	395	0.55	0.36	-0.77
18	G102	8.36	0.71	369	351	18	-0.09	0.73	-0.20	25	G316	5.06	0.48	635	239	396	-0.01	-0.91	-0.19
19	G396	8.36	0.35	172	153	19	0.33	-0.10	0.42	24	G306	5.01	0.85	789	392	397	0.68	-0.17	-0.44
20	G412	8.34	0.15	57	37	20	0.11	-0.26	0.54	23	G244	5.01	0.17	447	49	398	0.07	-0.48	-0.25
21	G227	8.32	0.35	171	150	21	0.25	0.35	0.50	22	G096	4.94	0.05	406	7	399	-0.09	0.11	-0.56
22	G418	8.30	0.37	196	174	22	0.27	0.34	0.39	21	G229	4.91	0.54	671	271	400	-0.29	-0.62	-0.26
23	G266	8.29	0.47	257	234	23	-0.10	-0.02	0.31	20	G285	4.84	0.52	663	262	401	-0.31	-0.45	-0.29
24	G400	8.27	0.36	188	164	24	0.34	0.06	0.51	19	G058	4.76	1.00	809	407	402	-0.30	-0.09	-0.27
25	G207	8.26	0.23	108	83	25	0.02	-0.08	0.29	18	G023	4.76	0.32	537	134	403	-0.72	-0.53	-0.31
26	G378	8.21	0.43	234	208	26.5	0.22	0.43	-0.08	17	G170	4.73	0.43	617	213	404	-0.23	-0.49	-0.65
27	G075	8.21	0.51	284	258	26.5	-0.18	0.36	-0.14	16	G338	4.61	0.36	570	165	405	-0.11	-0.46	-0.39
28	G383	8.20	0.37	201	173	28	0.33	0.33	0.48	15	G021	4.59	0.73	762	356	406	0.50	-0.58	-0.38
29	G391	8.18	0.33	177	148	29	0.42	-0.09	0.32	14	G045	4.56	0.79	784	377	407	0.35	-0.55	-0.38
30	G193	8.17	0.64	357	327	30	0.30	-0.05	0.09	13	G025	4.41	0.81	790	382	408	-0.59	-0.24	-0.29
31	G156	8.16	0.62	346	315	31	-0.23	0.55	0.33	12	G004	4.39	0.49	657	248	409	0.22	-0.44	-0.74
32	G184	8.15	0.43	243	211	32	-0.13	0.21	0.47	11	G282	4.29	1.14	823	413	410	0.69	-0.04	-0.89
33	G379	8.15	0.55	311	278	33	-0.03	0.39	0.20	10	G332	4.22	0.32	546	135	411	-0.17	-0.06	-0.86
34	G384	8.11	0.42	236	202	34	-0.01	0.41	-0.10	9	G287	4.04	0.53	676	264	412	0.19	-0.69	-0.47
35	G002	8.09	0.71	389	354	35	-0.50	0.09	-0.06	8	G294	3.95	0.43	622	209	413	0.19	-0.47	-0.46
36	G324	8.08	0.39	221	185	36	0.29	0.32	-0.45	7	G263	3.60	0.16	456	42	414	0.08	-0.38	-0.64
37	G326	8.06	0.64	362	325	37	0.08	-0.55	0.20	6	G275	3.55	0.55	695	280	415	0.36	-0.16	-0.61
38	G387	8.05	0.54	310	272	38	-0.39	0.02	0.38	5	G032	3.52	0.28	527	111	416	-0.11	-0.33	-0.72
39	G251	8.03	0.41	236	197	39	0.35	-0.04	0.26	4	G197	3.46	0.48	662	245	417	0.06	-0.73	-0.78
40	G292	8.02	1.41	458	418	40	0.99	0.06	0.22	3	G276	3.44	0.55	695	277	418	0.22	-0.41	-0.76
41	G410	8.01	0.03	46	5	41	0.19	-0.21	0.21	2	G196	3.39	0.38	597	178	419	0.14	-0.53	-0.74
42	G308	8.00	0.45	267	225	42	0.32	-0.10	0.33	1	G277	3.30	0.46	647	227	420	0.20	-0.12	-0.71

By definition, genotypes that appear close to the average environment axes (AEA) line (based on the average PC1 and PC2 scores of all locations) on the GGE biplot are considered stable across test sites. Whereas superior genotypes (high-yielding) are positioned on the right side along the AEA line. Accordingly, the GGE biplot determined G169, G420, G415, G139, G156, G412, G413, and G417 as superior genotypes (both high-yielding and stable) across the test sites; and G032, G282, G275, G276, G196, and G397 as the least performing genotypes (Fig 4A). As indicated by the double arrow line passing through AEA, the genotypes G077, G369, G085, G292, 389, and G397 were the most variable (highly unstable) genotypes with the highest GEI in terms of grain yield (Fig 4C). Based on double arrow line passing via AEA of GGE biplot for thousand kernel weight, G410, G418, and G088 were identified as stable and superior genotypes (Fig 4D), whereas G025, G058, G156, and G293 were highly unstable genotypes and had a higher contribution to the GEI (Fig 4D).

### Genotypic variation and genotype performances

The mean grain yield varied substantially among the evaluated genotypes, ranging from 3.3 to 9.8 t ha^-1^ (Table 4). The genotype G169, G413, G420, G415, G393, G390, G416, G417, G293, G139, and G253 were top-ranking in mean grain yield; whereas G277, G196, G276, G197, G032, G275, G263, G294, G287, G332, G282, and G004 were bottom-ranking (Table 4). As per the AMMI1analysis of grain yield, G369, G077, G397, G292 and G389 had higher IPCA1 value than the other genotypes and hence contributed more to the overall GEI. Whereas genotypes G169, G420 and G413 had the highest mean grain yield with lower IPCA1value (Table 5). Similarly, the AMMI1 of thousand kernel weight revealed that G126, G244, G418, G420, G405, G400, G353, G204, and G342 had higher mean performance than the overall mean performances of genotypes. Whereas, G025, G073, G276, G366, G156, G363, G355, and G287 had higher IPCA1 and hence had higher contribution to the overall GEI. Among the modern cultivars, more than half had low mean performance and wide adaptation across the test sites. According to the results revealed by AMMI and GGE-biplot, G169, G420, and G413 had the highest mean grain yield, whereas, G417, G411, G410, G412, G415 and G207 were the top-ranking stable genotypes with high grain yield (Table 5).

## Discussion

The ultimate goal of a plant breeder is to select desirable genotypes based on MET data and develop high-yielding cultivars with superior mean performance and stability across a wide range of environments [4, 6]. The use of suitable statistical models and tools helps to predict the mean performances of genotypes over multi-environments, thereby facilitating the selection of high- performing and broadly adapting genotypes [73–75]. However, such research work have been challenged by changing climate scenarios for decades and the future fate remains uncertain [10, 11]. The GEI is one of the key factors that affect cultivar improvement [70, 76] because it reduces the heritability of target traits in multi-environment trials [77]. In the developing world, farmers who lack or have limited access to agricultural inputs need stable cultivars for cultivation [23]. Hence, estimation of the complexity of GEI is necessary for the effective selection of genotypes with high and stable yield across multi-environment.

The results from the present study clearly emphasized the strongest effect of E, but also a significant effect from G and GEI on the mean performance of all measured phenotypic traits of durum wheat. Previous research indicated the comparable contribution of the G and E in determining almost any trait in wheat, and that the selection of G and E determines their relative importance [78]. However, despite the large E and GEI effects revealed in the present study, it was possible to identify stable and high-yielding genotypes in diverse environmets. In light of the predicted climate change, the stable productivity of crops will become an increasingly important factor in ensuring food security. The combined use of different multivariate statistical analyses on a broad range of genotypes grown in a variety of environments allows the detection of genotypes with desirable characteristics for use in plant breeding programs. This approach was used in the current study, which led to the identification of high-yielding and stable genotypes that could be used in durum wheat breeding programs for enhancing production and productivity.

Despite the significant effect of E, G, and GEI on the performance of the tested genotypes, E contributed the largest proportion of the total (G+E+GEI) variation for all traits except for the number of effective tillers per plant. This indicates that the test sites were significantly different, which affected the genotypes differently. Several factors contribute to the differences between test sites, including altitude, soil type, and the amount and distribution of rainfall. In line with this, the biplot analysis showed that Sinana and Holeta are distinct from Kulumsa, Chefe Donsa, and Akaki. The selection of genotypes and test locations play a significant role in the performances of durum wheat genotypes [78], and similar previous research also revealed the influence of test sites on the yield performance of durum wheat genotypes [16, 18, 41, 79–82].

In the present study, G was identified as the second-largest source of variation in most traits. This indicates that Ethiopian durum wheat gene pool has a high potential for the identification of desirable characteristics of these traits. Previous research reported G as an important contributor to phenotypic variations, while others found the G component as the most important among the sources of variation [83].

The present study clealy demonstrated that GEI significant contribution to the phenotypic variance, which suggests that genotypes performed differently in each environment and that their performance rank differ across test sites. The significant effect of GEI have been noted in several research reports on differents traits, including grain yield, in durum wheat landraces and cultivars [29, 80], in line with the resuls of the present study.

AMMI1 and AMMI2 biplots have been used in various previous studies to characterize and assess the relationship between E, G, and GEI [83–87]. In the present study, the AMMI analysis identified four stable and top-performing genotypes for each trait in each of the five test sites. Furthermore, the AMMI biplot analysis revealed G169, G420, G413, G139, G415, G416, G417, and G418 as high yielding and stable genotypes across test sites. Moreover, the results of AMMI stability values (ASV) and AMMI biplots were in good agreement, both predicted G169, G413, G420, G415, G393, G390, G416, G417, G293, G139, and G253 as high yielding and stable genotypes across the testing sites. Therefore, these genotypes are suitable for use in durum wheat breeding programs in order to develop new cultivars capable of producing high and stable grain yields in environments similar to those used in the present research. It is also desirable to use these genotypes along with other durum wheat germplasm in a genome-wide association study (GWAS) to identify quantitative trait loci (QTLs) associated with high and stable grain yields, potentially leading to the identification of alleles contributing to consistently high grain yields in durum wheat.

In AMMI2, the GEI decomposition displayed that IPCA1 and IPCA2 were highly significant (*P < 0*.*01*) for all traits, and the two PCs together explained more than 60% of the variance due to GEI. These results confirm reports from previous research [9, 28, 78] on the significance of GEI on the performance of genotypes across test sites. Testing sites with large IPCA1 and small IPCA2 scores are useful for discriminating and selecting against undesirable genotypes [35, 71]. The IPCA values obtained in the present study suggest a strong discriminating ability of Sinana for grain yield, spike length, and the number of effective tillers per plant, and hence can be used as a test site for selecting highly desirable genotypes for these traits. AMMI analysis showed that different genotypes performed differently in different environments for different traits. The results clearly demonstrate the need to understand the influence of environments on crop physiological traits and grain yield in order to have successful plant breeding programs, as indicated by previous studies [88, 89].

In the current study, we used a GGE biplot model to identify “winning” genotypes for all traits. As found in previous research [90, 91], the most suitable test site varied according to the traits evaluated, and that different environment-groups were optimal for different genotypes. According to Yan and Rajcan [71], a mega-environment is a group of homogeneous testing environments providing similar genotypic expression, thus assisting in selecting ideal genotypes and testing environments. According to “which won where” polygon view of the GGE-biplot of grain yield, G169, G420, G414, G413, and G394 performed better in the environment-groups comprising Kulumsa, Akaki, and Chefe Donsa, while G390, G393, and G292 performed better in the environment-group represented by Sinana. Hence, the use of these germplasm in durum wheat breeding programs is most appropriate when the areas targeted are those with similar environmental conditions.

In accordance with previous research [36, 87], the average-environment coordination (AEC) view of the GGE biplot was useful for assessing genotype performance and stability across test sites in this study. Likewise, as in prior research [70, 92], the arrow of the average environment axis (AEA), which points to genotypes with higher mean performance across test sites, was used to identify genotypes that are close to the ideal genotype in terms of performance and stability across the test sites. Given that G169 was at the position of the arrow of AEA in the center of the smallest concentric circle, it can be considered as ideal genotype for grain yield, whereas G415 and G420 were the closest to the ideal genotype, and hence is a highly desirable genotype that performs well along with the ideal genotype across test sites. Other superior genotypes both in grain yield and stability, as per the AEC view of the GGE biplot, were G413, G417, G156, G139, G102, G239, G326 and G412. The results indicate that these genotypes have a broad adaptation to diverse environments, and can therefore be used as candidates for further improvement through plant breeding. They can also be used in crossbreeding with other desirable genotypes for the development of superior cultivars with multiple desirable traits, including high and stable grain yield. Furthermore, the GGE biplot that displayed the discriminative ability and representativeness of test sites for grain yield indicated that Kulumsa, and Chefe Donsa were suitable test sites for selecting high yielding genotypes that are appropriate for use in the durum wheat breeding program in Ethiopia.

Using AMMI and GGE-biplot methods, the present study was able to identify high-yielding and stable durum wheat genotypes suitable to cultivation across major durum wheat-producing areas in Ethiopia. Both methods identified the same genotypes as superior (stable and high-yielding), which include G169, G115, and G139 among the landraces, and G417, and G420 among the cultivars. Despite this, there are some differences between the two approaches: G413, G416, G253, G393, G390, and G293 were among the superior genotypes according to AMMI, but G102 and G412 were among the superior genotypes according to the GGE biplot. This could be due to some differences in assumptions behind the AMMI and GGE biplot models for identifying high-yielding and stable genotypes [14]. Local adaptation was also noted for some of the genotypes through the AMMI analyses. For instance, G169, G420, G415, and G413 may be better suited to Kulumsa, Akaki, and Chefe Donsa, whereas G390, G393, and G292 may be best suited to Sinana. It is noteworthy that genotypes that are most stable and high-yielding across environments generally out-performed genotypes that are high-yielding in a particular test site or environment-group. Thus, given the findings of the present research, durum wheat breeding programs would benefit from strategies that promote the development of high-yielding cultivars that will be similarly adaptable across diverse agro-ecologies.

## Conclusions

This study revealed that GEI has a highly significant effect on the performance and stability of durum wheat genotypes. Hence, research activities aimed at identifying genotypes with high and stable yields for durum wheat breeding programs should seriously consider the effects of GEI. According to the results of this study, selecting genotypes that are suitable for breeding widely adaptable cultivars is a better strategy than selecting for breeding locally adapted cultivars. The present study clearly demonstrated the highly significant contribution of genotypic variance to the phenotypic variation in all traits studied. There is a high degree of genetic variation in the Ethiopian durum wheat gene pool; therefore, a subset of this diverse germplasm should be incorporated into breeding programs both locally and internationally for this crop. Both AMMI and GGE biplot identified two landraces (G169 and G139) and two cultivars (G417 and G420) as stable and high yielding across test sites. Therefore, the breeders should use these genotypes in the durum wheat improvement program to develop and deploy new stable and high-yielding cultivars in the environments represented by the five environments or similar in the respective target population of test sites. Through the application of an efficient breeding program that utilizes the Ethiopian durum wheat gene pool is insufficient, new cultivars adapted to climate change and global warming can be developed from these genetic materials, thereby contributing to food security in Ethiopia and beyond.

## Supporting information

S1 Table Lists of genotypes and combined mean performance of genotypes over test environments. (DOCX)
