# Supplementary material for: Multivariate analyses of Ethiopian durum wheat revealed stable and high yielding genotypes
Source: PLoS One. 2022 Aug 17;17(8):e0273008. doi: 10.1371/journal.pone.0273008 (PMC9385061; doi:10.1371/journal.pone.0273008)
Supplement: S1 Table — (DOCX) [file pone.0273008.s001.docx]

S1Table. Lists of genotypes and combined mean performance of genotypes for measured traits across test sites

| Genotype | AccN | DH | DM | SPPP | SPL | PLH | NET | STD | TGW | GYT |
| --- | --- | --- | --- | --- | --- | --- | --- | --- | --- | --- |
| G1 | 5328 | 74.53 | 136.15 | 17.23 | 7.1 | 96.49 | 6.34 | 85.47 | 39.61 | 6.32 |
| G2 | 5309 | 72.4 | 136.43 | 17.33 | 7.53 | 100.87 | 6.23 | 86.34 | 49.1 | 7.37 |
| G3 | 5332 | 71.42 | 136.5 | 16.89 | 7.79 | 95.89 | 6.16 | 84.93 | 41.71 | 6.46 |
| G4 | 5330 | 74.98 | 135.62 | 17.92 | 9.39 | 98.92 | 6.21 | 83.52 | 44.92 | 5.62 |
| G5 | 5087 | 75.01 | 136.32 | 18.13 | 8.6 | 98.11 | 6.13 | 85.43 | 42.32 | 6.42 |
| G6 | 5164 | 74.16 | 136.96 | 17.68 | 8.29 | 97.83 | 6.38 | 85.55 | 43.5 | 6.72 |
| G7 | 5001 | 73.22 | 136.19 | 17.43 | 8.82 | 96.96 | 6.32 | 86.16 | 44.95 | 6.2 |
| G8 | 5107 | 72.02 | 135.82 | 17.43 | 8.46 | 93.57 | 6.37 | 85.71 | 37.98 | 6.94 |
| G9 | 5109 | 73.52 | 136.42 | 18.54 | 8.33 | 98.2 | 6.26 | 85.9 | 48.27 | 6.66 |
| G10 | 5204 | 71.72 | 135.52 | 16.79 | 8.4 | 99.01 | 6.18 | 85.13 | 42.61 | 6.57 |
| G11 | 5228 | 75.14 | 135.94 | 18.12 | 8.81 | 100.92 | 6.08 | 84.45 | 42.46 | 7.03 |
| G12 | 5473 | 73.53 | 135.95 | 18.07 | 7.96 | 101.15 | 6.35 | 84.3 | 38.91 | 6.91 |
| G13 | 5257 | 71.27 | 135.13 | 17.47 | 8.14 | 91.34 | 6.17 | 84.06 | 41.75 | 6.79 |
| G14 | 5256 | 73.57 | 136.16 | 17.71 | 8.21 | 93.91 | 6.28 | 86.43 | 39.68 | 6.72 |
| G15 | 5259 | 71.81 | 136.38 | 17.09 | 7.55 | 97.97 | 6.27 | 84.74 | 47.3 | 6.98 |
| G16 | 5255 | 71.68 | 135.62 | 18.35 | 8.36 | 104.07 | 6.18 | 85.14 | 41.63 | 6.91 |
| G17 | 5168 | 72.15 | 135.67 | 17.42 | 7.66 | 94.61 | 6.24 | 85.43 | 39.56 | 7.37 |
| G18 | 5254 | 74.07 | 135.52 | 17.41 | 6.98 | 97.93 | 6.01 | 84.92 | 40.37 | 6.54 |
| G19 | 5014 | 70.55 | 135.53 | 18.05 | 7.46 | 97.84 | 6.32 | 85.41 | 42.76 | 6.97 |
| G20 | 5020 | 70.2 | 135.13 | 17.43 | 9.21 | 96.64 | 6.24 | 85.56 | 40 | 7.22 |
| G21 | 5468 | 74.56 | 135.82 | 17.57 | 8.05 | 96.77 | 6.1 | 85.12 | 43.97 | 5.86 |
| G22 | 5471 | 73.64 | 135.89 | 17.73 | 7.25 | 103.94 | 6.06 | 84.35 | 39.08 | 6.23 |
| G23 | 5260 | 73.26 | 135.92 | 17.24 | 7.19 | 96.11 | 6.19 | 86.03 | 39.33 | 6.09 |
| G24 | 5261 | 72.98 | 135.61 | 16.8 | 7.82 | 95.06 | 6.36 | 84.99 | 38.27 | 5.98 |
| G25 | 5338 | 74.14 | 136 | 17.89 | 7.16 | 92.45 | 6.15 | 86.07 | 35.27 | 5.83 |
| G26 | 5336 | 71.51 | 135.98 | 16.63 | 8.22 | 99.03 | 6.15 | 84.69 | 38.94 | 6.46 |
| G27 | 5056 | 70.68 | 135.18 | 17.72 | 9.51 | 96.93 | 6.34 | 85.27 | 38.21 | 6.95 |
| G28 | 5291 | 74.51 | 136.17 | 17.99 | 7.78 | 98.7 | 6.31 | 85.5 | 39.05 | 6.57 |
| G29 | 5343 | 71.45 | 135.92 | 17.25 | 8.28 | 99.24 | 6.21 | 84.93 | 36.06 | 6.43 |
| G30 | 5601 | 71.67 | 135.88 | 18.12 | 7.86 | 97.35 | 6.1 | 85.76 | 38.57 | 6.8 |
| G31 | 5267 | 74.24 | 135.96 | 17.8 | 8.41 | 95.82 | 6.44 | 85.41 | 37.5 | 6.7 |
| G32 | 5163 | 75.01 | 137.86 | 17.56 | 9.37 | 113.42 | 6.05 | 84.51 | 36.17 | 5.37 |
| G33 | 5246 | 73.53 | 136.1 | 17.35 | 6.8 | 96.53 | 6.07 | 85.76 | 40.43 | 6.37 |
| G34 | 5192 | 73.43 | 135.94 | 17.71 | 7.28 | 102 | 6.31 | 85.57 | 39.86 | 6.99 |
| G35 | 5071 | 72.93 | 135.74 | 17.72 | 8.39 | 97.2 | 6.17 | 85.15 | 47.41 | 7.32 |
| G36 | 5245 | 72.18 | 136.5 | 18.2 | 8.55 | 91.04 | 6.35 | 84.09 | 38.54 | 7.05 |
| G37 | 5070 | 71.65 | 135.77 | 17.42 | 8.54 | 92.63 | 6.13 | 83.75 | 39.13 | 6.89 |
| G38 | 5242 | 72.68 | 136.1 | 17.38 | 7.96 | 99.48 | 6.24 | 83.53 | 42.09 | 6.56 |
| G39 | 5023 | 75.78 | 135.99 | 18.05 | 7.9 | 92.81 | 6.33 | 85.31 | 35.64 | 7.05 |
| G40 | 5388 | 72.72 | 136.9 | 18.78 | 8.62 | 98.79 | 6.19 | 85.58 | 44.66 | 7.68 |
| G41 | 5078 | 73.5 | 136.21 | 16.94 | 6.97 | 96.97 | 6.12 | 85.44 | 44.71 | 7.14 |
| G42 | 5348 | 70.52 | 136.01 | 16.43 | 8.17 | 96.43 | 6.22 | 83.86 | 40.88 | 6.28 |
| G43 | 5344 | 75.57 | 136.87 | 18.07 | 8.45 | 94.61 | 6.25 | 86.27 | 38.89 | 7.48 |
| G44 | 5342 | 73.9 | 136.24 | 17.68 | 8.55 | 98.14 | 6.3 | 85.42 | 43.77 | 6.84 |
| G45 | 5340 | 73.63 | 136.16 | 17.99 | 7.54 | 103.73 | 6.15 | 84.9 | 37.6 | 5.79 |

Where AccN: Accession name, DH: Days to heading, DM: Days to maturity, SPPP: Number of spikelets per spike, SPL: Spike length (cm), PLH: Plant height (cm), NET: Tillers per plant, STD: Stand percent per plot, TKW: Thousand-kernel weight (gm), GYD: Grain yield (t ha^-1^)

S1Table. Lists of genotypes and combined mean performance of genotypes for measured traits across test sites

| Genotype | AccN | DH | DM | SPPP | SPL | PLH | NET | STD | TGW | GYT |
| --- | --- | --- | --- | --- | --- | --- | --- | --- | --- | --- |
| G46 | 5052 | 72.03 | 135.7 | 17.14 | 8.01 | 98.23 | 6.04 | 84.21 | 39.94 | 6.28 |
| G47 | 5049 | 72.6 | 135.7 | 17.8 | 6.99 | 97.07 | 6.14 | 86.19 | 40.64 | 6.49 |
| G48 | 5051 | 73.01 | 136.34 | 17.12 | 8.22 | 101.38 | 6.13 | 84.85 | 44.9 | 6.21 |
| G49 | 5207 | 70.5 | 135.69 | 17.26 | 7.88 | 98.67 | 6.22 | 84.02 | 39.83 | 6.26 |
| G50 | 5623 | 72.14 | 135.4 | 17.1 | 7.62 | 95.09 | 6.23 | 85.11 | 37.1 | 6.23 |
| G51 | 5558 | 73.49 | 136.08 | 17.25 | 7.06 | 98.14 | 6.11 | 84.99 | 37.93 | 6.74 |
| G52 | 5504 | 74.34 | 135.79 | 17.48 | 8.4 | 95.66 | 6.1 | 85.34 | 37.86 | 6.52 |
| G53 | 5188 | 74.69 | 135.74 | 17.53 | 7.86 | 92.57 | 6.21 | 85.29 | 39.85 | 6.5 |
| G54 | 5278 | 72.46 | 135.96 | 17 | 8.49 | 96.59 | 6.16 | 84.83 | 38.11 | 7.19 |
| G55 | 5281 | 69.55 | 135.59 | 17.71 | 7.89 | 96.05 | 5.96 | 85.14 | 40.75 | 6.71 |
| G56 | 5627 | 73.7 | 136.13 | 18.15 | 7.98 | 96.92 | 6.12 | 84.54 | 39.04 | 6.62 |
| G57 | 5554 | 73.3 | 136.64 | 17.54 | 7.18 | 98.17 | 6.31 | 85.61 | 40.81 | 6.63 |
| G58 | 5516 | 76.37 | 138.72 | 18.34 | 7.78 | 99.29 | 6.27 | 83.92 | 39.31 | 5.89 |
| G59 | 5026 | 72.73 | 136.71 | 16.97 | 8.26 | 97.67 | 6.26 | 85.1 | 41.62 | 6.6 |
| G60 | 5057 | 71.06 | 135.34 | 17.28 | 9.3 | 92.81 | 6.22 | 84.52 | 37.93 | 7.05 |
| G61 | 5104 | 71.03 | 135.51 | 17.02 | 7.32 | 100.7 | 6.11 | 85.09 | 49.36 | 6.72 |
| G62 | 5288 | 72.68 | 135.81 | 18.05 | 9.07 | 97.26 | 6.18 | 85.76 | 36.04 | 6.53 |
| G63 | 5617 | 70.86 | 136.29 | 18.08 | 8.96 | 99.24 | 6.38 | 84.94 | 41.14 | 7.22 |
| G64 | 5216 | 71.24 | 135.67 | 17.44 | 9.26 | 97.9 | 6.16 | 84.93 | 39.8 | 6.54 |
| G65 | 5577 | 75.69 | 136.01 | 18.29 | 8.77 | 98.24 | 6.07 | 85.32 | 41.77 | 6.24 |
| G66 | 5482 | 74.54 | 136.04 | 17.19 | 6.83 | 97.28 | 6.13 | 85.34 | 40.41 | 6.55 |
| G67 | 5573 | 75 | 135.95 | 17.58 | 7.21 | 96.16 | 6.21 | 84.51 | 34.13 | 5.96 |
| G68 | 5552 | 71.61 | 135.67 | 17.84 | 8.53 | 97.87 | 6.19 | 84.93 | 42.28 | 6.94 |
| G69 | 5236 | 74.3 | 136.37 | 17.9 | 8.17 | 96.22 | 6.35 | 85.26 | 41.76 | 7.04 |
| G70 | 5467 | 71.13 | 135.09 | 17.72 | 8.85 | 97.33 | 6.2 | 85.68 | 38.38 | 7.3 |
| G71 | 5009 | 72.53 | 136.31 | 18.05 | 7.83 | 98.39 | 6.02 | 84.42 | 45.47 | 6.5 |
| G72 | 5383 | 73.42 | 135.98 | 17.86 | 9.25 | 100.68 | 6.14 | 84.9 | 41.36 | 6.88 |
| G73 | 5162 | 71.74 | 135.68 | 17.36 | 7.05 | 98.75 | 6.11 | 86.09 | 38.24 | 6.42 |
| G74 | 5006 | 71.61 | 135.93 | 17.36 | 8.08 | 98.98 | 6.13 | 84.63 | 44.03 | 6.26 |
| G75 | 5169 | 74.14 | 135.5 | 17.95 | 8.73 | 95.34 | 6.25 | 85.63 | 42.03 | 7.65 |
| G76 | 5576 | 72.72 | 136.08 | 17.26 | 7.46 | 98.58 | 6.23 | 84.54 | 41.5 | 6.49 |
| G77 | 5181 | 73.82 | 136.5 | 17.62 | 8.75 | 96.66 | 6.02 | 84.54 | 43.58 | 6.96 |
| G78 | 5239 | 75.3 | 135.58 | 17.81 | 8.64 | 95.3 | 6.16 | 84.66 | 39.22 | 6.95 |
| G79 | 5375 | 76.08 | 136.42 | 18.26 | 7.67 | 94.95 | 6.19 | 84.87 | 44.73 | 6.37 |
| G80 | 5574 | 71.98 | 135.82 | 17.71 | 8.91 | 94.44 | 6.18 | 85.87 | 42.41 | 7.1 |
| G81 | 5561 | 74.43 | 135.97 | 17.68 | 7.26 | 91.01 | 6.16 | 86.57 | 41.81 | 6.5 |
| G82 | 5476 | 71.11 | 135.42 | 17.4 | 7.98 | 96.86 | 6.24 | 85.97 | 39.24 | 6.95 |
| G83 | 5215 | 73.14 | 135.99 | 18.16 | 9.03 | 96.63 | 6 | 85.83 | 44.04 | 7.26 |
| G84 | 5294 | 71.12 | 135.68 | 17.48 | 7.6 | 97.44 | 6.11 | 85.45 | 41.8 | 6.87 |
| G85 | 5286 | 75.53 | 136.5 | 17.42 | 8.21 | 96.2 | 6.41 | 85.67 | 40.88 | 6.68 |
| G86 | 5452 | 72.94 | 136.09 | 18.03 | 8.14 | 97.97 | 6.21 | 85.02 | 36.24 | 6.78 |
| G87 | 5451 | 74.63 | 136.38 | 17.8 | 7.17 | 96.07 | 6.26 | 85.02 | 41.85 | 7.03 |
| G88 | 5364 | 72.51 | 135.12 | 17.68 | 7.85 | 96.76 | 6.47 | 86.06 | 39.21 | 7.35 |
| G89 | 5365 | 70.02 | 135.19 | 17.32 | 8.59 | 99.89 | 6.35 | 84.81 | 41.12 | 6.69 |
| G90 | 5220 | 73.65 | 135.8 | 18.22 | 9.18 | 98.15 | 6.21 | 83.9 | 38.01 | 6.98 |

Where AccN: Accession name, DH: Days to heading, DM: Days to maturity, SPPP: Number of spikelets per spike, SPL: Spike length (cm), PLH: Plant height (cm), NET: Tillers per plant, STD: Stand percent per plot, TKW: Thousand-kernel weight (gm), GYD: Grain yield (t ha^-1^)

S1Table. Lists of genotypes and combined mean performance of genotypes for measured traits across test sites

| Genotype | AccN | DH | DM | SPPP | SPL | PLH | NET | STD | TGW | GYT |
| --- | --- | --- | --- | --- | --- | --- | --- | --- | --- | --- |
| G91 | 5519 | 74.51 | 135.77 | 16.75 | 8.21 | 95.34 | 6.5 | 84.13 | 40.3 | 6.25 |
| G92 | 5523 | 71.86 | 135.49 | 17.33 | 7.85 | 94.88 | 6.3 | 85.79 | 38.03 | 6.15 |
| G93 | 5202 | 70.77 | 135.58 | 17.42 | 8.92 | 96.75 | 6.15 | 84.75 | 39.16 | 6.59 |
| G94 | 5522 | 72.73 | 136.2 | 17.2 | 7.64 | 97.17 | 6.28 | 84.76 | 38.51 | 6.47 |
| G95 | 5182 | 73.81 | 136.13 | 18.13 | 8.59 | 97.92 | 6.12 | 85.49 | 43.97 | 6.83 |
| G96 | 5174 | 72.79 | 136.03 | 17.72 | 6.91 | 98.94 | 6.18 | 85.39 | 41.06 | 5.98 |
| G97 | 5579 | 73.57 | 135.94 | 17.03 | 7.73 | 94.52 | 6.06 | 85.31 | 40.88 | 6.61 |
| G98 | 5397 | 74.7 | 136.23 | 17.87 | 7.07 | 95.3 | 6.06 | 86.25 | 42.37 | 6.75 |
| G99 | 5526 | 74.66 | 135.88 | 17.74 | 7.72 | 93.08 | 6.16 | 84.45 | 39.75 | 6.47 |
| G100 | 5410 | 74.11 | 136.16 | 17.73 | 7.4 | 97.9 | 6.03 | 85.69 | 42.3 | 7.23 |
| G101 | 5414 | 70.82 | 135.45 | 17.74 | 8.02 | 97.74 | 6.21 | 85.04 | 41.97 | 6.58 |
| G102 | 5146 | 71.86 | 135.55 | 18.01 | 8.57 | 96.28 | 6.26 | 86.35 | 38.59 | 7.31 |
| G103 | 5155 | 76.93 | 136.8 | 18.84 | 9.41 | 94.8 | 6.24 | 86.31 | 38.46 | 7.3 |
| G104 | 5041 | 71.25 | 135.51 | 17.82 | 9.15 | 97.97 | 6.24 | 85.59 | 39.83 | 6.94 |
| G105 | 5572 | 74.45 | 136.2 | 17.45 | 7.92 | 94.96 | 6.18 | 84.85 | 40.27 | 6.3 |
| G106 | 5453 | 73.87 | 136.1 | 17.41 | 7.46 | 91.82 | 6.14 | 85.82 | 38.26 | 7.11 |
| G107 | 5102 | 74.63 | 135.77 | 17.61 | 8.08 | 94.09 | 6.26 | 85.84 | 42.07 | 7.37 |
| G108 | 5420 | 76.8 | 136.43 | 18.11 | 8.22 | 94.37 | 6.19 | 84.82 | 36.98 | 5.82 |
| G109 | 5119 | 65.11 | 135.03 | 16.4 | 7.99 | 98.82 | 6.17 | 83.85 | 38.71 | 6.44 |
| G110 | 5422 | 73.68 | 136.14 | 17.62 | 8.34 | 93.52 | 6.03 | 84.74 | 42.15 | 6.75 |
| G111 | 5583 | 74.36 | 136.17 | 17.95 | 8.38 | 93.99 | 6.28 | 85.2 | 39.39 | 6.51 |
| G112 | 5136 | 67.95 | 135.11 | 16.89 | 7.85 | 94.02 | 6.12 | 85.17 | 44.85 | 6.63 |
| G113 | 5117 | 75.21 | 136.33 | 18.06 | 6.88 | 100.64 | 6.15 | 85.26 | 43.07 | 6.73 |
| G114 | 5612 | 74.06 | 136.32 | 17.56 | 7.22 | 96.17 | 6.1 | 85.76 | 42.34 | 6.69 |
| G115 | 5158 | 72.91 | 135.93 | 18.25 | 8.51 | 96.66 | 6.11 | 85.56 | 44.74 | 6.67 |
| G116 | 5459 | 72.65 | 136.09 | 17.49 | 7.95 | 97.99 | 6.13 | 85.05 | 37.71 | 6.78 |
| G117 | 5394 | 74.1 | 136.38 | 17.66 | 7.77 | 96.62 | 6.3 | 84.35 | 42.7 | 6.67 |
| G118 | 5393 | 71.57 | 136.44 | 18.14 | 7.87 | 95.21 | 6.09 | 85.01 | 43.2 | 6.56 |
| G119 | 5142 | 73.34 | 136.59 | 16.98 | 7.51 | 96.28 | 6.23 | 86.51 | 43.11 | 7.05 |
| G120 | 5327 | 69.34 | 135.64 | 17.02 | 7.63 | 94.51 | 6.32 | 84.28 | 44.2 | 6.36 |
| G121 | 5172 | 74.26 | 135.62 | 17.98 | 7.68 | 91.81 | 6.17 | 85.15 | 35.84 | 6.4 |
| G122 | 5610 | 73.67 | 135.74 | 17.47 | 7.84 | 98.43 | 6.3 | 86.1 | 43.26 | 7.26 |
| G123 | 5602 | 72.22 | 136.27 | 17.14 | 6.85 | 93.73 | 6.14 | 84.42 | 40.44 | 6.56 |
| G124 | 5305 | 74.32 | 135.62 | 17.51 | 7.92 | 94.63 | 6.19 | 84.27 | 35.27 | 6.82 |
| G125 | 5371 | 73.29 | 135.66 | 17.13 | 7.78 | 92.85 | 6.38 | 84.96 | 40.06 | 6.86 |
| G126 | 5326 | 72.81 | 135.99 | 17.47 | 7.96 | 92.03 | 6.17 | 84.73 | 37.91 | 6.84 |
| G127 | 5544 | 73.99 | 136.2 | 17.93 | 8.27 | 98.77 | 6.18 | 85.84 | 39.3 | 7.01 |
| G128 | 5299 | 71.86 | 135.76 | 17.2 | 8.23 | 99.34 | 6.41 | 84.66 | 41.71 | 6.67 |
| G129 | 5219 | 73.55 | 135.85 | 17.92 | 8.01 | 94.92 | 6.18 | 84.37 | 39.09 | 6.55 |
| G130 | 5098 | 75.09 | 136.4 | 17.49 | 7.98 | 95.89 | 6.21 | 85.22 | 45.71 | 7.02 |
| G131 | 5306 | 73.4 | 135.74 | 17.78 | 7.41 | 98.36 | 6.21 | 85.29 | 38.97 | 6.5 |
| G132 | 5159 | 70.96 | 135.66 | 17.68 | 7.75 | 100.02 | 6.15 | 85.31 | 46.75 | 6.55 |
| G133 | 5183 | 72.31 | 136.16 | 17.68 | 8.43 | 96.9 | 6.11 | 84.99 | 40.74 | 6.78 |
| G134 | 5184 | 73.14 | 136.27 | 17.37 | 7.79 | 93.29 | 6.42 | 85.4 | 40.01 | 7.16 |
| G135 | 5048 | 73.36 | 136.49 | 18.27 | 8.49 | 97.25 | 6.21 | 84.78 | 43.46 | 7.03 |

Where AccN: Accession name, DH: Days to heading, DM: Days to maturity, SPPP: Number of spikelets per spike, SPL: Spike length (cm), PLH: Plant height (cm), NET: Tillers per plant, STD: Stand percent per plot, TKW: Thousand-kernel weight (gm), GYD: Grain yield (t ha^-1^)

S1Table. Lists of genotypes and combined mean performance of genotypes for measured traits across test sites

| Genotype | AccN | DH | DM | SPPP | SPL | PLH | NET | STD | TGW | GYT |
| --- | --- | --- | --- | --- | --- | --- | --- | --- | --- | --- |
| G136 | 5140 | 70.46 | 136.19 | 15.92 | 7.75 | 97.02 | 6.21 | 83.82 | 40.41 | 5.95 |
| G137 | 7077 | 70.65 | 135.76 | 17.79 | 8.59 | 95.87 | 6.36 | 85.85 | 40.99 | 7.32 |
| G138 | 5465 | 69.21 | 136.02 | 17.95 | 7.71 | 97.83 | 6.17 | 84.78 | 40.48 | 6.32 |
| G139 | 5076 | 71.81 | 135.62 | 18.01 | 8.5 | 96.54 | 6.04 | 86.12 | 39.26 | 7.52 |
| G140 | 5017 | 74.93 | 136.15 | 17.6 | 7.2 | 94.67 | 6.17 | 85.28 | 40.33 | 6.78 |
| G141 | 5005 | 73.53 | 135.92 | 17.1 | 8.63 | 96.33 | 6.09 | 84.65 | 39.69 | 6.52 |
| G142 | 5062 | 74.22 | 136.01 | 17.76 | 6.88 | 97.82 | 6.08 | 85.33 | 38.47 | 6.46 |
| G143 | 5591 | 74.91 | 135.41 | 18.01 | 8.4 | 95.45 | 6.26 | 84.78 | 40.71 | 6.56 |
| G144 | 5073 | 67.64 | 135.39 | 16.72 | 8.11 | 96.01 | 6.22 | 86.04 | 38.11 | 6.36 |
| G145 | 5191 | 70.46 | 135.42 | 17.41 | 9.29 | 101.75 | 6.37 | 84.87 | 47.16 | 7.25 |
| G146 | 5461 | 74.03 | 136.1 | 17.25 | 7.9 | 97.33 | 6.22 | 84.92 | 39.31 | 6.64 |
| G147 | 5551 | 75.9 | 135.7 | 17.38 | 8.59 | 95.06 | 6.16 | 84.16 | 38.76 | 6.51 |
| G148 | 5597 | 72.96 | 136.47 | 17.14 | 7.45 | 94.34 | 6.08 | 85.66 | 43.31 | 7.15 |
| G149 | 5518 | 73 | 135.85 | 17.86 | 8.93 | 96.47 | 6.3 | 85.55 | 43.47 | 6.84 |
| G150 | 5047 | 70.34 | 135.1 | 17.2 | 8.46 | 97.15 | 6.08 | 85.34 | 39.24 | 6.88 |
| G151 | 5213 | 73.97 | 136.55 | 17.73 | 7.73 | 94.51 | 6.28 | 85.43 | 39.91 | 7.06 |
| G152 | 5241 | 72.44 | 136.18 | 17.57 | 7.63 | 100.6 | 6.14 | 86.05 | 37.7 | 6.46 |
| G153 | 5165 | 73.75 | 136.12 | 17.55 | 7.25 | 95.78 | 6.18 | 85.48 | 40.12 | 6.49 |
| G154 | 5011 | 73.16 | 136.08 | 17.64 | 8.34 | 95.84 | 6.19 | 85.09 | 45.34 | 7.08 |
| G155 | 5039 | 74.46 | 135.34 | 18.69 | 8.82 | 94.29 | 6.25 | 86.09 | 41.85 | 6.81 |
| G156 | 5433 | 73.15 | 135.78 | 18.17 | 7.07 | 97.5 | 6.03 | 86.55 | 41.58 | 7.52 |
| G157 | 5571 | 72.09 | 135.66 | 17.64 | 8.65 | 98.59 | 6.21 | 85.4 | 41.95 | 6.94 |
| G158 | 5401 | 75.49 | 136.87 | 18.5 | 8.25 | 95.35 | 6.32 | 85.21 | 44.15 | 6.36 |
| G159 | 5177 | 70.44 | 135.61 | 18.03 | 7.27 | 99.96 | 6.23 | 86.13 | 41.71 | 7.06 |
| G160 | 5230 | 73.25 | 135.79 | 18.33 | 7.28 | 97.78 | 6.07 | 87.14 | 40.06 | 7.09 |
| G161 | 5289 | 76.05 | 136.08 | 18.06 | 7.48 | 95.12 | 5.96 | 85.82 | 37.36 | 6.65 |
| G162 | 5426 | 67.03 | 135.88 | 16.83 | 7.78 | 94.91 | 6.25 | 84.44 | 38.09 | 6.3 |
| G163 | 5272 | 73.57 | 136.15 | 17.03 | 7.32 | 95.13 | 6.21 | 85.22 | 47.18 | 6.84 |
| G164 | 5095 | 70.08 | 136.62 | 16.85 | 7.15 | 96.25 | 6.21 | 84.71 | 49.08 | 7.3 |
| G165 | 5372 | 74.39 | 135.97 | 18.28 | 8.81 | 95.55 | 6.16 | 85.02 | 41.18 | 6.68 |
| G166 | 5015 | 70.36 | 135.84 | 16.66 | 7.51 | 96.31 | 6.25 | 85.1 | 47.55 | 6.62 |
| G167 | 5434 | 70.62 | 135.98 | 17.4 | 7.4 | 100.13 | 6.22 | 85.87 | 42.85 | 6.9 |
| G168 | 5249 | 74.99 | 137.22 | 17.29 | 6.94 | 97.43 | 6.09 | 85.76 | 39.53 | 6.6 |
| G169 | 5189 | 73.4 | 136.18 | 17.65 | 8.71 | 95.63 | 6.19 | 85.58 | 40.36 | 8.01 |
| G170 | 5066 | 75.78 | 136.71 | 17.3 | 8.77 | 103.47 | 6.16 | 84.68 | 38.81 | 5.64 |
| G171 | 5190 | 74.33 | 136.31 | 17.93 | 8.51 | 97.89 | 6.32 | 85.95 | 38.69 | 6.73 |
| G172 | 5025 | 72.4 | 136.37 | 17.69 | 8.46 | 103.35 | 6.01 | 85.64 | 48.94 | 6.41 |
| G173 | 5562 | 72.61 | 136.78 | 17.44 | 7.89 | 97.21 | 6.01 | 84.75 | 40.26 | 7.04 |
| G174 | 5251 | 71.27 | 135.81 | 17.86 | 8.5 | 98.86 | 6.16 | 86.22 | 40.61 | 7.31 |
| G175 | 5030 | 72.44 | 135.83 | 17.77 | 8.3 | 94.88 | 6.19 | 85.48 | 38.93 | 6.58 |
| G176 | 5563 | 74.6 | 136.48 | 17.33 | 6.84 | 94.75 | 6.16 | 85.04 | 40.24 | 6.66 |
| G177 | 5457 | 73.47 | 136.49 | 17.87 | 7.24 | 97.05 | 6.04 | 84.49 | 42.2 | 7.01 |
| G178 | 5506 | 74.72 | 136.34 | 17.5 | 7.94 | 97.99 | 6.15 | 85.27 | 37.44 | 6.47 |
| G179 | 5018 | 75.46 | 136.25 | 17.64 | 8.43 | 96.15 | 6.28 | 84.95 | 36.43 | 6.77 |
| G180 | 5035 | 73.46 | 136.17 | 17.97 | 8.91 | 97.87 | 6.33 | 86.09 | 44.52 | 7.38 |

Where AccN: Accession name, DH: Days to heading, DM: Days to maturity, SPPP: Number of spikelets per spike, SPL: Spike length (cm), PLH: Plant height (cm), NET: Tillers per plant, STD: Stand percent per plot, TKW: Thousand-kernel weight (gm), GYD: Grain yield (t ha^-1^)

S1Table. Lists of genotypes and combined mean performance of genotypes for measured traits across test sites

| Genotype | AccN | DH | DM | SPPP | SPL | PLH | NET | STD | TGW | GYT |
| --- | --- | --- | --- | --- | --- | --- | --- | --- | --- | --- |
| G181 | 5234 | 74.98 | 136.17 | 18.29 | 8.31 | 99.45 | 6.13 | 85.74 | 43.68 | 6.5 |
| G182 | 5125 | 73.34 | 136.44 | 17.34 | 7.72 | 97.13 | 6.26 | 84.97 | 36.37 | 6.26 |
| G183 | 5543 | 72.46 | 135.78 | 17.71 | 7.83 | 98.53 | 6.44 | 85.63 | 46.59 | 6.98 |
| G184 | 5065 | 71.8 | 136.07 | 17.1 | 7.97 | 94.43 | 6 | 85.13 | 42.39 | 7.4 |
| G185 | 5594 | 74.76 | 135.97 | 16.93 | 7.53 | 92.95 | 6.4 | 85.9 | 38.83 | 6.49 |
| G186 | 5619 | 71.8 | 135.13 | 17.67 | 7.9 | 93.73 | 6.2 | 85.13 | 39.18 | 6.66 |
| G187 | 5069 | 68.54 | 135.28 | 17 | 8.63 | 96.02 | 6.22 | 85.9 | 40.63 | 6.96 |
| G188 | 5387 | 72.97 | 135.71 | 18.46 | 8.22 | 94.75 | 6.05 | 85.51 | 40.14 | 6.87 |
| G189 | 5100 | 73.65 | 136.03 | 17.77 | 8.21 | 95.69 | 6.29 | 84.7 | 40.99 | 6.82 |
| G190 | 5565 | 74.53 | 136.45 | 18.35 | 8.93 | 96.05 | 6.25 | 85.4 | 42.43 | 6.98 |
| G191 | 5567 | 72.09 | 136.04 | 18.08 | 7.8 | 99.86 | 6.03 | 85.52 | 38.91 | 6.48 |
| G192 | 5432 | 70.45 | 135.42 | 17.69 | 8.3 | 97.85 | 6.41 | 84.94 | 39.21 | 7.42 |
| G193 | 2211 | 71.45 | 135.45 | 17.13 | 8.21 | 96.67 | 6.07 | 85.87 | 37.85 | 7.65 |
| G194 | 4649 | 71.03 | 136.29 | 17.64 | 7.77 | 95.42 | 6.17 | 84.95 | 43.77 | 6.8 |
| G195 | 5020 | 70.52 | 135.57 | 17.39 | 8.48 | 94.84 | 6.18 | 85.06 | 37.47 | 6.64 |
| G196 | 5043 | 75.58 | 138.66 | 16.1 | 8.17 | 105.96 | 6.11 | 83.01 | 37.77 | 5.03 |
| G197 | 5044 | 75.89 | 139.17 | 16.6 | 8.21 | 106.35 | 6.16 | 83.39 | 37.44 | 5.15 |
| G198 | 5057 | 70.81 | 135.41 | 17.14 | 8.32 | 94.29 | 6.28 | 84.23 | 37.53 | 6.85 |
| G199 | 5140 | 74.07 | 136.09 | 17.64 | 8.51 | 92.82 | 6.16 | 85.11 | 42.37 | 6.73 |
| G200 | 5141 | 74.37 | 136.09 | 17.55 | 6.65 | 96.26 | 6.1 | 85.33 | 36.14 | 6.5 |
| G201 | 5142 | 76.13 | 136.79 | 17.59 | 7.42 | 93.9 | 6.04 | 85.54 | 38.03 | 6.53 |
| G202 | 5143 | 77.38 | 136.71 | 18.76 | 8.93 | 94.92 | 6.13 | 86.26 | 37.29 | 7.14 |
| G203 | 5149 | 71.83 | 135.64 | 17.86 | 9 | 96.59 | 6.25 | 85.28 | 40.13 | 7.38 |
| G204 | 5152 | 69.07 | 135.73 | 17.62 | 8.12 | 97.7 | 6.05 | 84.81 | 48.9 | 6.55 |
| G205 | 5158 | 72.59 | 135.96 | 17.84 | 8.41 | 95.56 | 6.08 | 86.03 | 42.06 | 6.72 |
| G206 | 5168 | 73.78 | 135.85 | 17.46 | 7.42 | 95.15 | 6.3 | 85.71 | 38.94 | 6.95 |
| G207 | 5169 | 72.42 | 135.23 | 17.6 | 8.8 | 98.77 | 6.44 | 85.21 | 41.18 | 7.63 |
| G208 | 5171 | 75.01 | 135.39 | 17.35 | 9.08 | 97.48 | 6.16 | 84.39 | 39.52 | 6.96 |
| G209 | 3540 | 66.89 | 134.58 | 17.73 | 8.25 | 93.49 | 6.12 | 84.34 | 41.28 | 6.47 |
| G210 | 5179 | 75.47 | 136.27 | 17.31 | 6.8 | 91.93 | 5.84 | 84.4 | 40.09 | 6.5 |
| G211 | 5181 | 73.69 | 136.02 | 17.94 | 8.43 | 97.43 | 6.12 | 85.14 | 44.24 | 6.91 |
| G212 | 5182 | 74.04 | 135.56 | 18.23 | 8.81 | 96.9 | 6.36 | 85.06 | 39.96 | 7.03 |
| G213 | 5183 | 73.52 | 135.42 | 17.56 | 8.38 | 96.38 | 6.06 | 84.88 | 40.57 | 7.02 |
| G214 | 5197 | 70.82 | 136.01 | 17.33 | 7.91 | 99.25 | 6.26 | 84.15 | 41.04 | 6.71 |
| G215 | 5198 | 70.41 | 136.09 | 17.4 | 7.96 | 98.98 | 6.07 | 84.94 | 41.41 | 6.35 |
| G216 | 5214 | 73.25 | 136.22 | 17.51 | 7.79 | 94.89 | 6.17 | 84.34 | 35.54 | 6.48 |
| G217 | 5342 | 75.33 | 136.36 | 17.3 | 8.02 | 96.5 | 6.06 | 84.89 | 45.06 | 7.09 |
| G218 | 5344 | 74.75 | 136.39 | 17.56 | 7.11 | 95.45 | 6.08 | 85.24 | 38.99 | 6.11 |
| G219 | 5354 | 71.07 | 136.53 | 17.65 | 6.87 | 99.08 | 6.15 | 85.91 | 36.98 | 6.14 |
| G220 | 5369 | 74.18 | 136.65 | 17.45 | 6.85 | 94.61 | 6.17 | 84.87 | 39.87 | 6.98 |
| G221 | 5373 | 75.48 | 135.53 | 17.64 | 8.27 | 93.28 | 6.17 | 86.71 | 35.66 | 6.98 |
| G222 | 5434 | 72.41 | 136.74 | 17.62 | 8.39 | 96.58 | 6.2 | 84.49 | 43.86 | 7.18 |
| G223 | 5441 | 73.51 | 136.92 | 17.84 | 7.72 | 97.43 | 6.14 | 84.85 | 40.06 | 6.29 |
| G224 | 5465 | 68.36 | 135.35 | 16.62 | 7.47 | 99.81 | 6.22 | 85.38 | 40.26 | 6.49 |
| G225 | 5470 | 72.45 | 136.32 | 17.62 | 7.75 | 93.5 | 6.19 | 84.98 | 35.15 | 6.21 |

Where AccN: Accession name, DH: Days to heading, DM: Days to maturity, SPPP: Number of spikelets per spike, SPL: Spike length (cm), PLH: Plant height (cm), NET: Tillers per plant, STD: Stand percent per plot, TKW: Thousand-kernel weight (gm), GYD: Grain yield (t ha^-1^)

S1Table. Lists of genotypes and combined mean performance of genotypes for measured traits across test sites

| Genotype | AccN | DH | DM | SPPP | SPL | PLH | NET | STD | TGW | GYT |
| --- | --- | --- | --- | --- | --- | --- | --- | --- | --- | --- |
| G226 | 5472 | 73.79 | 136.2 | 17.76 | 7.35 | 95.68 | 6.02 | 86.11 | 40.54 | 6.57 |
| G227 | 5491 | 71.36 | 135.85 | 17.45 | 8.27 | 95.15 | 6.29 | 85.07 | 39.47 | 7.61 |
| G228 | 5492 | 74.31 | 135.64 | 17.48 | 7.84 | 95.65 | 6.18 | 84.67 | 39.55 | 6.73 |
| G229 | 5502 | 74.04 | 136.24 | 16.92 | 7.05 | 95.89 | 6.12 | 84.94 | 36.61 | 5.79 |
| G230 | 5504 | 74.15 | 136.22 | 17.68 | 8.48 | 94.69 | 6.04 | 85.53 | 41.85 | 7.08 |
| G231 | 5507 | 74.57 | 135.95 | 17.29 | 7.86 | 95.45 | 6.31 | 85.56 | 38.27 | 6.86 |
| G232 | 5510 | 75.1 | 136.21 | 17.36 | 6.83 | 93.16 | 6.21 | 84.65 | 37.12 | 6.22 |
| G233 | 5515 | 67.19 | 135.5 | 18.15 | 8.66 | 100.34 | 5.97 | 85.69 | 45.77 | 6.89 |
| G234 | 5526 | 73.62 | 135.69 | 17.23 | 8.14 | 96.93 | 6.18 | 85.06 | 40.72 | 6.71 |
| G235 | 5537 | 71.65 | 135.83 | 16.99 | 8.41 | 98.42 | 6.12 | 85.19 | 42.54 | 7.24 |
| G236 | 5572 | 74.06 | 136.67 | 17.45 | 7.72 | 101.33 | 6.27 | 84.33 | 40.63 | 6.11 |
| G237 | 5576 | 75.08 | 136.47 | 17.73 | 6.97 | 92.31 | 6.15 | 85.56 | 37.97 | 6.12 |
| G238 | 7019 | 72.37 | 136.41 | 17.85 | 8.7 | 94.74 | 6.25 | 84.5 | 39.86 | 7.11 |
| G239 | 5581 | 76.45 | 136.39 | 18.18 | 7.55 | 93.91 | 6.23 | 84.74 | 38.71 | 6.75 |
| G240 | 5582 | 74.33 | 137.55 | 17.91 | 8.58 | 97.23 | 6.16 | 85.71 | 44.45 | 7.22 |
| G241 | 5586 | 73.05 | 136.27 | 17.35 | 8.38 | 96.58 | 6.15 | 83.9 | 40.63 | 6.7 |
| G242 | 5591 | 72.97 | 136.47 | 17.72 | 7.48 | 99.3 | 6.01 | 85.23 | 39.66 | 6.55 |
| G243 | 5593 | 73.28 | 135.25 | 17.91 | 8.55 | 97.14 | 6.15 | 86 | 40.3 | 7.05 |
| G244 | 5597 | 74.24 | 135.4 | 17.61 | 7.66 | 94.26 | 6.22 | 85.43 | 36.91 | 5.61 |
| G245 | 5600 | 69.22 | 135.27 | 17.43 | 8.68 | 99.87 | 6.06 | 85.31 | 46.73 | 7.18 |
| G246 | 5609 | 71.32 | 135.62 | 17.4 | 7.73 | 90.61 | 6.15 | 85.04 | 36.61 | 6.31 |
| G247 | 5618 | 73.96 | 136.09 | 17.84 | 8.77 | 95.02 | 6.22 | 85.37 | 44.65 | 7.12 |
| G248 | 5627 | 74.55 | 136.04 | 17.38 | 8.01 | 95.41 | 6.12 | 86 | 40.48 | 6.67 |
| G249 | 5642 | 74.52 | 136.23 | 17.93 | 7.79 | 95.03 | 6.21 | 85.83 | 39.89 | 6.5 |
| G250 | 5653 | 72 | 135.71 | 18.2 | 8.48 | 100.56 | 6.16 | 86.17 | 44.84 | 7.42 |
| G251 | 5666 | 73.6 | 135.69 | 17.45 | 8.06 | 95.77 | 6.22 | 84.81 | 39.3 | 7.18 |
| G252 | 5669 | 74.36 | 136.07 | 17.22 | 7.2 | 98.3 | 6.15 | 85.69 | 39.77 | 6.68 |
| G253 | 5707 | 74.89 | 136.55 | 18.09 | 8.41 | 96.03 | 6.1 | 85.52 | 44.21 | 7.35 |
| G254 | 5729 | 70.82 | 135.42 | 18.02 | 9.15 | 94.69 | 6.26 | 84.9 | 36.39 | 6.48 |
| G255 | 5892 | 70.17 | 135.66 | 17.49 | 7.8 | 99.09 | 6.1 | 84.65 | 42.1 | 6.92 |
| G256 | 5893 | 73.81 | 135.69 | 17.9 | 7.71 | 94.37 | 6.29 | 86.27 | 35.3 | 6.91 |
| G257 | 5898 | 73.78 | 136.29 | 17.28 | 7.6 | 94.28 | 6.07 | 85.36 | 40.72 | 6.7 |
| G258 | 5909 | 72.85 | 135.88 | 17.11 | 7.99 | 93.61 | 6.15 | 85.82 | 38.97 | 6.82 |
| G259 | 5485 | 74.49 | 136.79 | 18.24 | 8.11 | 96.24 | 6.11 | 85.85 | 43.37 | 6.42 |
| G260 | 5103 | 73.91 | 135.82 | 18.42 | 8.22 | 96.62 | 6.02 | 85.62 | 47.88 | 6.59 |
| G261 | 5923 | 73.67 | 135.71 | 17.86 | 8.66 | 95.83 | 6.12 | 85.89 | 44.94 | 6.95 |
| G262 | 6102 | 69.92 | 135.17 | 17.73 | 8.81 | 95.28 | 6.18 | 86.19 | 40.23 | 7.33 |
| G263 | 6914 | 73.23 | 139.4 | 16.02 | 7.93 | 105.76 | 6 | 83.89 | 39.74 | 5.32 |
| G264 | 6933 | 72.05 | 135.38 | 17.63 | 7.32 | 98.13 | 6.16 | 85.24 | 41.38 | 6.5 |
| G265 | 6936 | 72.71 | 135.7 | 16.93 | 7.61 | 98.36 | 6.21 | 85.39 | 49.74 | 6.75 |
| G266 | 6955 | 73.79 | 135.73 | 17.19 | 7.75 | 92.96 | 6.14 | 84.58 | 37.35 | 7.5 |
| G267 | 6968 | 74.05 | 135.52 | 17.35 | 7.37 | 89.75 | 5.95 | 85.05 | 35.99 | 6.37 |
| G268 | 6971 | 74.1 | 136 | 17.18 | 8.31 | 90.66 | 6.16 | 84.49 | 37.87 | 6.38 |
| G269 | 6974 | 75.04 | 135.29 | 17.58 | 7.8 | 92.08 | 6.07 | 83.67 | 38.09 | 6.38 |
| G270 | 6975 | 72.91 | 136.09 | 17.48 | 8.24 | 93.65 | 6.17 | 85.2 | 36.95 | 7.39 |

Where AccN: Accession name, DH: Days to heading, DM: Days to maturity, SPPP: Number of spikelets per spike, SPL: Spike length (cm), PLH: Plant height (cm), NET: Tillers per plant, STD: Stand percent per plot, TKW: Thousand-kernel weight (gm), GYD: Grain yield (t ha^-1^)

S1Table. Lists of genotypes and combined mean performance of genotypes for measured traits across test sites

| Genotype | AccN | DH | DM | SPPP | SPL | PLH | NET | STD | TGW | GYT |
| --- | --- | --- | --- | --- | --- | --- | --- | --- | --- | --- |
| G271 | 6983 | 74.12 | 135.61 | 17.48 | 8.53 | 93.07 | 6.12 | 84.84 | 38.85 | 6.62 |
| G272 | 6987 | 75.24 | 137.16 | 18.65 | 9.23 | 92.92 | 6.28 | 85.66 | 36.17 | 7.18 |
| G273 | 6988 | 74.3 | 136.73 | 17.35 | 8.43 | 99.06 | 6.1 | 85.8 | 43.69 | 7.15 |
| G274 | 5568 | 75.38 | 136.29 | 18.14 | 8.14 | 100.58 | 6.18 | 84.67 | 40.82 | 6.06 |
| G275 | 7000 | 75.12 | 138.83 | 17.13 | 8.38 | 105.7 | 6.22 | 84.03 | 38.36 | 5.36 |
| G276 | 7002 | 74.66 | 139.52 | 16.42 | 8.25 | 103.57 | 6.17 | 83.79 | 37.66 | 5.22 |
| G277 | 7003 | 74.31 | 139.25 | 16.33 | 8.01 | 106.2 | 6.16 | 84.34 | 37.12 | 5.26 |
| G278 | 7004 | 72.58 | 135.96 | 17.72 | 8.52 | 95.67 | 6.15 | 85.5 | 39.94 | 7.09 |
| G279 | 7007 | 70.48 | 135.42 | 17.82 | 8.72 | 96.12 | 6.09 | 85.72 | 44.02 | 7.44 |
| G280 | 7009 | 71.38 | 135.09 | 17.91 | 8.44 | 94.19 | 6.23 | 86.27 | 36.24 | 7 |
| G281 | 7010 | 72.78 | 135.93 | 17.68 | 8.61 | 99.1 | 6.19 | 85.75 | 38.24 | 7.12 |
| G282 | 7014 | 75.05 | 138.65 | 16.57 | 8.07 | 108.35 | 6.1 | 84.7 | 39.54 | 5.56 |
| G283 | 7015 | 69.82 | 134.95 | 17.49 | 8.62 | 93.9 | 6.18 | 85.83 | 40.19 | 7.19 |
| G284 | 7018 | 69.1 | 135.12 | 17.31 | 8.85 | 93.91 | 6.2 | 86.08 | 39.42 | 6.66 |
| G285 | 7020 | 69.93 | 135.81 | 17.78 | 7.93 | 99.66 | 6.31 | 85.52 | 38.61 | 5.93 |
| G286 | 7031 | 70.82 | 135.4 | 17.11 | 8.27 | 95.9 | 6.14 | 85.49 | 37.31 | 6.93 |
| G287 | 5548 | 75.91 | 138.47 | 16.71 | 8.34 | 106.74 | 6.29 | 84.21 | 38.2 | 5.48 |
| G288 | 7046 | 68.29 | 135.56 | 17.78 | 8.03 | 93.93 | 6.14 | 85.9 | 36.2 | 6.49 |
| G289 | 7050 | 74.38 | 135.87 | 18.43 | 8.96 | 96.9 | 6.15 | 85.5 | 38.21 | 6.69 |
| G290 | 7056 | 71.65 | 136.08 | 17.55 | 8.47 | 92.41 | 6.21 | 85.23 | 38.75 | 6.88 |
| G291 | 7060 | 71.07 | 135.32 | 17.08 | 8.19 | 94.71 | 6.15 | 84.47 | 38.3 | 6.56 |
| G292 | 7063 | 72.09 | 135.37 | 17.34 | 8.71 | 94.99 | 6.29 | 86.13 | 39.08 | 7.6 |
| G293 | 7064 | 69.83 | 135.14 | 17.38 | 8.88 | 95.34 | 6.15 | 84.4 | 38.62 | 7.9 |
| G294 | 7069 | 75.64 | 138.8 | 16.4 | 8.11 | 103.78 | 6.11 | 83.89 | 40.02 | 5.48 |
| G295 | 7075 | 69.04 | 135.73 | 17.12 | 8.54 | 94.92 | 6.26 | 84.69 | 40.49 | 6.57 |
| G296 | 7076 | 70.58 | 135.61 | 17.35 | 8.34 | 94.44 | 6.18 | 84.1 | 39.09 | 6.82 |
| G297 | 7078 | 69.17 | 135.54 | 16.87 | 8.63 | 97.01 | 6.33 | 85.76 | 39.07 | 6.56 |
| G298 | 7082 | 72.4 | 136.84 | 18.03 | 9 | 95.34 | 6.02 | 84.52 | 44.03 | 6.9 |
| G299 | 7083 | 73.44 | 136.66 | 17.82 | 9.02 | 97.03 | 6.21 | 85.17 | 38.78 | 6.48 |
| G300 | 7084 | 75.6 | 137.91 | 18.82 | 8.62 | 93.2 | 6.2 | 85.43 | 38.71 | 7.39 |
| G301 | 7104 | 71.65 | 136.02 | 17.14 | 7.56 | 99.66 | 6.05 | 85.58 | 43.18 | 6.44 |
| G302 | 7133 | 75.23 | 136.58 | 19.15 | 8.71 | 95 | 6.18 | 85.86 | 36.47 | 6.44 |
| G303 | 7135 | 74.55 | 136.98 | 18.19 | 8.74 | 92.31 | 6.22 | 84.56 | 40.11 | 6.89 |
| G304 | 7150 | 75.54 | 136.32 | 19.19 | 8.84 | 95.53 | 6.24 | 85.54 | 38.7 | 6.87 |
| G305 | 7201 | 74.84 | 135.57 | 18.16 | 8.3 | 95.36 | 6.29 | 85.73 | 39.02 | 6.67 |
| G306 | 7205 | 77.28 | 137.42 | 18.81 | 8.93 | 95.31 | 6.22 | 84.84 | 40.95 | 5.9 |
| G307 | 7207 | 74.29 | 136.18 | 18.1 | 9.38 | 95.6 | 6.37 | 85.31 | 36.16 | 7.04 |
| G308 | 7209 | 72.54 | 135.34 | 18.58 | 9.2 | 95.28 | 6.38 | 86.54 | 38.76 | 7.35 |
| G309 | 7210 | 73.3 | 136.35 | 18.12 | 7.07 | 101 | 6.13 | 85.96 | 40.51 | 6.56 |
| G310 | 7218 | 73.86 | 136.8 | 18.1 | 7.41 | 97.01 | 6.12 | 85.65 | 39.65 | 6.31 |
| G311 | 7242 | 74.45 | 136.06 | 16.89 | 6.76 | 94.14 | 6 | 84.85 | 40.75 | 6.76 |
| G312 | 7295 | 72.13 | 135.84 | 17.7 | 8.03 | 94.44 | 6.23 | 84.52 | 37.91 | 6.52 |
| G313 | 7313 | 68.21 | 135.44 | 17.07 | 8.21 | 96.58 | 6.78 | 84.88 | 41.39 | 6.79 |
| G314 | 7317 | 68.49 | 135.83 | 16.89 | 7.96 | 97.22 | 6.07 | 84.67 | 42.02 | 6.8 |
| G315 | 7343 | 76.89 | 136.81 | 18.83 | 8.53 | 93.52 | 6.24 | 84.66 | 38.74 | 6.95 |

Where AccN: Accession name, DH: Days to heading, DM: Days to maturity, SPPP: Number of spikelets per spike, SPL: Spike length (cm), PLH: Plant height (cm), NET: Tillers per plant, STD: Stand percent per plot, TKW: Thousand-kernel weight (gm), GYD: Grain yield (t ha^-1^)

S1Table. Lists of genotypes and combined mean performance of genotypes for measured traits across test sites

| Genotype | AccN | DH | DM | SPPP | SPL | PLH | NET | STD | TGW | GYT |
| --- | --- | --- | --- | --- | --- | --- | --- | --- | --- | --- |
| G316 | 7375 | 74.54 | 138.89 | 16.64 | 8.41 | 105.73 | 5.99 | 83.93 | 40.27 | 5.61 |
| G317 | 7378 | 69.42 | 135.66 | 17.05 | 6.88 | 91.39 | 6.1 | 83.99 | 44.62 | 7.06 |
| G318 | 7464 | 73.02 | 136.22 | 16.77 | 7.52 | 96.55 | 6.01 | 84.08 | 46.1 | 6.7 |
| G319 | 7477 | 70.46 | 135.8 | 16.8 | 7.74 | 95.04 | 6.04 | 84.79 | 41.55 | 6.5 |
| G320 | 5269 | 72.15 | 135.93 | 16.55 | 7.47 | 97.1 | 5.97 | 85.5 | 47.68 | 7.14 |
| G321 | 7532 | 70.47 | 136.03 | 17.19 | 7.41 | 100.77 | 6.19 | 84.62 | 45.56 | 6.2 |
| G322 | 7568 | 71.82 | 136.19 | 17.69 | 8.51 | 95.19 | 6.19 | 86.28 | 40.37 | 7 |
| G323 | 7569 | 73.22 | 135.89 | 17.08 | 7.82 | 93.22 | 6.25 | 85.45 | 36.03 | 7.05 |
| G324 | 7572 | 72.18 | 135.77 | 17.34 | 7.7 | 96.04 | 6.06 | 84.12 | 36.95 | 7.11 |
| G325 | 7576 | 72.82 | 136.07 | 16.59 | 7.84 | 96.89 | 6.08 | 84.54 | 46.44 | 6.42 |
| G326 | 7578 | 74.65 | 136.89 | 17.44 | 8.18 | 97.75 | 6.29 | 85.83 | 37.44 | 7.36 |
| G327 | 7580 | 75.47 | 136.05 | 17.33 | 7.88 | 95.86 | 6.05 | 85.29 | 38.44 | 6.83 |
| G328 | 7581 | 73.64 | 136.15 | 17.62 | 8.33 | 98.33 | 6.15 | 86.1 | 37.66 | 7.26 |
| G329 | 7626 | 75.33 | 136.13 | 17.65 | 8.18 | 96.71 | 6.26 | 86.3 | 36.1 | 6.58 |
| G330 | 7629 | 70.52 | 136.39 | 17.64 | 8.24 | 96.36 | 6.15 | 84.97 | 41.21 | 6.57 |
| G331 | 7631 | 71.52 | 136.55 | 18.04 | 7.96 | 96.28 | 6.13 | 84.19 | 42.41 | 6.89 |
| G332 | 7641 | 71.69 | 135.92 | 15.34 | 7.67 | 96.16 | 6.14 | 82.57 | 36.46 | 5.47 |
| G333 | 7647 | 72.79 | 135.97 | 17.53 | 7.96 | 93.61 | 6.03 | 86.1 | 40.3 | 6.93 |
| G334 | 7649 | 74.6 | 136.85 | 17.93 | 7.29 | 95.67 | 6 | 84.92 | 39.32 | 6.48 |
| G335 | 7664 | 72.63 | 136.33 | 17.68 | 8.23 | 94.74 | 6.11 | 85.14 | 37.6 | 6.37 |
| G336 | 7666 | 72.5 | 135.72 | 17.29 | 7.12 | 89.75 | 6.21 | 85.78 | 36.74 | 6.74 |
| G337 | 7673 | 73.68 | 135.92 | 17.5 | 8.16 | 93.71 | 6.15 | 85.63 | 41.22 | 6.85 |
| G338 | 7683 | 72.95 | 136.3 | 17.15 | 7.93 | 92.86 | 6.16 | 84.53 | 39.73 | 5.71 |
| G339 | 7710 | 71.08 | 135.59 | 17.16 | 8.62 | 96.61 | 6.25 | 85.91 | 41.06 | 6.99 |
| G340 | 7712 | 73.15 | 136.52 | 18.52 | 8.78 | 90.63 | 6.22 | 84.76 | 38.33 | 6.88 |
| G341 | 7713 | 75.27 | 136.89 | 16.8 | 8.1 | 95.86 | 6.08 | 85.08 | 38.73 | 6.95 |
| G342 | 7798 | 73.02 | 136.4 | 17.62 | 8.93 | 100.3 | 6.16 | 86.16 | 40.58 | 7.01 |
| G343 | 7801 | 73.12 | 135.24 | 17.97 | 7.97 | 98.48 | 6.08 | 85.81 | 37.84 | 7.18 |
| G344 | 7822 | 73.96 | 135.44 | 17.48 | 7.39 | 93.45 | 6.22 | 85.98 | 36.32 | 6.99 |
| G345 | 7823 | 72.62 | 134.7 | 17.23 | 7.51 | 93.14 | 6.19 | 85.02 | 34.41 | 6.97 |
| G346 | 3540 | 73.98 | 135.9 | 17.81 | 8.05 | 94.78 | 6.2 | 85.21 | 37.04 | 6.59 |
| G347 | 7826 | 73.71 | 135.84 | 17.84 | 8.21 | 97.94 | 6.2 | 85 | 36.7 | 6.31 |
| G348 | 7827 | 73.65 | 135.08 | 17.7 | 7.96 | 96.36 | 6.19 | 84.43 | 37.7 | 6.99 |
| G349 | 7828 | 74 | 136.24 | 18.17 | 8.15 | 99.52 | 6.21 | 85.84 | 45.8 | 7.33 |
| G350 | 7832 | 73.57 | 136.35 | 16.81 | 7.14 | 90.64 | 6.09 | 83.49 | 36.43 | 5.92 |
| G351 | 7880 | 73.54 | 136.15 | 18.23 | 8.54 | 97.48 | 6.18 | 84.84 | 44.2 | 7.25 |
| G352 | 7999 | 75.29 | 136.01 | 17.78 | 7.56 | 93.18 | 6.1 | 84.82 | 41.53 | 6.73 |
| G353 | 8072 | 70.57 | 135.53 | 16.57 | 7.45 | 97.86 | 6.09 | 85.36 | 48.11 | 6.59 |
| G354 | 214370 | 74.81 | 135.4 | 16.37 | 8.61 | 96.57 | 6.09 | 84.9 | 43.4 | 5.91 |
| G355 | 222393 | 75.76 | 137.47 | 17.77 | 7.36 | 99.17 | 6.05 | 83.97 | 45.78 | 6.25 |
| G356 | 222427 | 74.1 | 136.21 | 17.2 | 7.31 | 98.6 | 6.17 | 85.06 | 35.88 | 6.34 |
| G357 | 226225 | 74.04 | 137.1 | 17.96 | 7.23 | 99.55 | 6.03 | 85.72 | 42.78 | 6.42 |
| G358 | 226241 | 73.38 | 136.37 | 17.4 | 6.88 | 94.02 | 6.14 | 84.8 | 39.2 | 6.91 |
| G359 | 7832 | 75.12 | 136.53 | 17.96 | 7.77 | 96.13 | 6.16 | 85.01 | 38.94 | 7.28 |
| G360 | 226897 | 74.05 | 136.24 | 18.3 | 8.7 | 95.35 | 6.27 | 86.4 | 39.28 | 7.28 |

Where AccN: Accession name, DH: Days to heading, DM: Days to maturity, SPPP: Number of spikelets per spike, SPL: Spike length (cm), PLH: Plant height (cm), NET: Tillers per plant, STD: Stand percent per plot, TKW: Thousand-kernel weight (gm), GYD: Grain yield (t ha^-1^)

S1Table. Lists of genotypes and combined mean performance of genotypes for measured traits across test sites

| Genotype | AccN | DH | DM | SPPP | SPL | PLH | NET | STD | TGW | GYT |
| --- | --- | --- | --- | --- | --- | --- | --- | --- | --- | --- |
| G361 | 230678 | 74.08 | 136.19 | 17.66 | 7.96 | 93.73 | 6.1 | 85.53 | 39.91 | 7.09 |
| G362 | 235051 | 67.36 | 135.37 | 16.13 | 8.02 | 98.19 | 6.23 | 84.54 | 34.82 | 5.87 |
| G363 | 238891 | 71.6 | 136.28 | 17.13 | 7.73 | 100.3 | 6.13 | 85 | 45.37 | 6.66 |
| G364 | 239693 | 69.03 | 135.4 | 16.94 | 8.12 | 94.09 | 6.33 | 85.63 | 40.05 | 7.04 |
| G365 | 239694 | 69.01 | 135.81 | 16.54 | 8.12 | 100.32 | 6.35 | 85.08 | 48.7 | 6.62 |
| G366 | 239711 | 68.68 | 136.05 | 17.11 | 8.14 | 100.37 | 6.12 | 84.4 | 46.35 | 6.91 |
| G367 | 242779 | 68.56 | 135.34 | 17.76 | 9 | 95.81 | 6.21 | 84.46 | 40.57 | 7.01 |
| G368 | 242781 | 69.07 | 136.1 | 16.75 | 7.68 | 87.48 | 6 | 86.07 | 47.78 | 6.78 |
| G369 | 242782 | 74.18 | 136.1 | 17.71 | 7.72 | 97.18 | 6.11 | 84.73 | 46.2 | 6.47 |
| G370 | 242783 | 72.02 | 136.51 | 17.4 | 8.54 | 99.45 | 6.22 | 85.17 | 44.86 | 6.93 |
| G371 | 242784 | 74.33 | 136.43 | 17.7 | 7.24 | 95.36 | 6.21 | 86 | 40.25 | 7.59 |
| G372 | 242785 | 73.81 | 135.97 | 18.29 | 8.81 | 100.4 | 6.17 | 86.2 | 40.95 | 6.76 |
| G373 | 242786 | 73.33 | 136.04 | 18.05 | 9.26 | 97.93 | 6.25 | 84.99 | 42.72 | 6.95 |
| G374 | 242787 | 74.98 | 136.86 | 17.51 | 7.45 | 93.33 | 6.17 | 85.91 | 40.74 | 7 |
| G375 | 242789 | 74.29 | 135.42 | 17.39 | 8.06 | 95.14 | 6.25 | 84.54 | 35.99 | 6.46 |
| G376 | 242790 | 75.33 | 136.5 | 17.82 | 7.06 | 93.46 | 6.11 | 85.94 | 40.84 | 7.19 |
| G377 | 242791 | 69.9 | 135.8 | 17.64 | 8.09 | 93.11 | 5.99 | 85.3 | 43.36 | 7.23 |
| G378 | 242792 | 73.59 | 136.27 | 17.64 | 6.45 | 98.94 | 6.14 | 86.16 | 38.56 | 7.46 |
| G379 | 242793 | 74.71 | 135.65 | 18.03 | 8.35 | 94.23 | 5.96 | 84.05 | 41.44 | 7.27 |
| G380 | 243698 | 73.63 | 135.65 | 17.21 | 6.61 | 97.43 | 6.15 | 86.4 | 38.8 | 6.46 |
| G381 | 243700 | 72.96 | 135.76 | 17.31 | 8.41 | 94.16 | 6.09 | 85.37 | 36.78 | 6.64 |
| G382 | 243701 | 70.15 | 135.47 | 16.81 | 7.74 | 94.74 | 6.19 | 85.22 | 37.3 | 6.24 |
| G383 | 243703 | 75.53 | 135.89 | 17.65 | 8.6 | 98 | 6.21 | 86.09 | 38.22 | 7.36 |
| G384 | 243706 | 75.71 | 135.43 | 18.27 | 9 | 94.08 | 6.1 | 85.85 | 36.35 | 6.99 |
| G385 | 243717 | 75.17 | 136.46 | 18.35 | 9.14 | 99.46 | 6.07 | 85.15 | 38.52 | 6.77 |
| G386 | 274497 | 74.83 | 135.79 | 17.11 | 7.7 | 97.33 | 6.08 | 85 | 43.52 | 6.2 |
| G387 | Yerer | 70.39 | 136.18 | 17.43 | 7.69 | 87.63 | 6.23 | 86.06 | 48.02 | 7.29 |
| G388 | Kilinto | 68.53 | 135.69 | 16.93 | 6.7 | 100.84 | 5.99 | 84.13 | 45.83 | 6.57 |
| G389 | Quamy | 68.35 | 136.2 | 16.79 | 6.38 | 100.13 | 6.05 | 83.93 | 49.1 | 6.27 |
| G390 | Mangudo | 68.22 | 135.86 | 17.77 | 7.18 | 100.6 | 6.05 | 85.1 | 48.83 | 7.45 |
| G391 | Mukiye | 68.58 | 135.94 | 16.99 | 6.37 | 90.39 | 6.13 | 84.17 | 46.83 | 7.22 |
| G392 | Ginchi | 65.15 | 135.78 | 17.06 | 7.24 | 100.94 | 6.11 | 83.65 | 48.89 | 7.47 |
| G393 | Dembi | 68.7 | 135.87 | 16.76 | 6.5 | 88.95 | 6.17 | 85.41 | 40.91 | 7.75 |
| G394 | Bichena | 66.25 | 135.94 | 17.09 | 6.58 | 100.18 | 5.98 | 83.89 | 46.64 | 6.55 |
| G395 | Robe | 66.96 | 136.17 | 16.31 | 6.37 | 98 | 6.16 | 84.21 | 46.85 | 6.67 |
| G396 | Don-matteo | 70.92 | 135.68 | 16.79 | 6.58 | 83.19 | 6.05 | 84.01 | 47.6 | 7.45 |
| G397 | Alem-tena | 67.91 | 136.21 | 16.59 | 5.98 | 83.52 | 6.09 | 83.76 | 40.63 | 6.77 |
| G398 | Ld-357 | 70.49 | 135.97 | 17.44 | 6.98 | 99.5 | 6.23 | 85 | 39.19 | 6.31 |
| G399 | Tesfaye | 70.91 | 135.73 | 17.38 | 6.5 | 84.56 | 6.05 | 84.03 | 39.69 | 7.13 |
| G400 | Werer | 69.18 | 136.5 | 17.09 | 6.36 | 88.04 | 6.18 | 84.87 | 49.15 | 7.3 |
| G401 | Hitosa | 68.52 | 135.99 | 17.08 | 7.03 | 88.58 | 5.96 | 84.64 | 40.13 | 6.65 |
| G402 | Utuba | 67.45 | 136.5 | 17.05 | 6.44 | 87.01 | 6.02 | 85.01 | 48.61 | 6.84 |
| G403 | Gerado | 69.04 | 136.63 | 17.48 | 8.2 | 90.37 | 6.02 | 85.09 | 47.39 | 6.29 |
| G404 | Assasa | 69.01 | 135.99 | 17.63 | 7.99 | 97.48 | 6.12 | 85.48 | 43.69 | 7.33 |
| G405 | Arandato | 72.96 | 135.42 | 17.91 | 7.32 | 95.64 | 6.15 | 85.46 | 36.04 | 6.13 |

Where AccN: Accession name, DH: Days to heading, DM: Days to maturity, SPPP: Number of spikelets per spike, SPL: Spike length (cm), PLH: Plant height (cm), NET: Tillers per plant, STD: Stand percent per plot, TKW: Thousand-kernel weight (gm), GYD: Grain yield (t ha^-1^)

S1Table. Lists of genotypes and combined mean performance of genotypes for measured traits across test sites

| Genotype | AccN | DH | DM | SPPP | SPL | PLH | NET | STD | TGW | GYT |
| --- | --- | --- | --- | --- | --- | --- | --- | --- | --- | --- |
| G406 | Tob-66 | 66.44 | 135.59 | 17.02 | 6.82 | 99.96 | 5.97 | 83.21 | 48.98 | 6.21 |
| G407 | Boohai | 67.68 | 136.01 | 16.83 | 6.98 | 103.31 | 6.06 | 83.34 | 47.95 | 6.39 |
| G408 | Cocorit-71 | 67.79 | 135.54 | 16.99 | 6.48 | 87.99 | 6.02 | 85.06 | 42.5 | 6.89 |
| G409 | Foka | 68.34 | 135.84 | 17.07 | 6.58 | 103.35 | 6.09 | 84.16 | 43.02 | 6.19 |
| G410 | Ude | 68.58 | 135.5 | 16.33 | 6.19 | 87 | 6.17 | 84.63 | 49.65 | 7.08 |
| G411 | Bakalcha | 67.74 | 135.97 | 16.43 | 6.61 | 86.64 | 6.01 | 84.98 | 46.22 | 7.03 |
| G412 | Ejersa | 70.39 | 136.32 | 16.98 | 7.51 | 86.92 | 6.03 | 84.45 | 44.9 | 7.2 |
| G413 | Tate | 68.21 | 135.98 | 16.13 | 6.71 | 87.94 | 6.13 | 86.16 | 41.13 | 7.83 |
| G414 | Oda | 67.9 | 136.35 | 17.2 | 7.13 | 97.62 | 6.1 | 85.37 | 42.36 | 6.63 |
| G415 | Toltu | 69.72 | 135.47 | 16.66 | 6.27 | 83.28 | 6.02 | 85.43 | 39.86 | 7.82 |
| G416 | Ebsa | 68.99 | 135.53 | 16.39 | 6.42 | 83.7 | 6.14 | 84.84 | 41.78 | 7.5 |
| G417 | Dire | 69.61 | 135.81 | 16.93 | 7.11 | 85.78 | 6 | 85.06 | 43.35 | 7.25 |
| G418 | Ilani | 66.94 | 135.31 | 16.77 | 7.8 | 94.39 | 6.12 | 83.97 | 50.41 | 7.15 |
| G419 | Leliso | 66.88 | 136.05 | 17.49 | 6.63 | 100.79 | 6.17 | 84.14 | 45.1 | 6.97 |
| G420 | Bulala | 69.08 | 135.39 | 16.98 | 7.2 | 92.13 | 6 | 84.98 | 44.95 | 7.7 |

Where AccN: Accession name, DH: Days to heading, DM: Days to maturity, SPPP: Number of spikelets per spike, SPL: Spike length (cm), PLH: Plant height (cm), NET: Tillers per plant, STD: Stand percent per plot, TKW: Thousand-kernel weight (gm), GYD: Grain yield (t ha^-1^)
